# E-Cannula reveals anatomical diversity in sharp-wave ripples as a driver for the recruitment of distinct hippocampal assemblies

**DOI:** 10.1016/j.celrep.2022.111453

**Published:** 2022-10-04

**Authors:** Xin Liu, Satoshi Terada, Mehrdad Ramezani, Jeong-Hoon Kim, Yichen Lu, Andres Grosmark, Attila Losonczy, Duygu Kuzum

**Affiliations:** 1 Department of Electrical and Computer Engineering, University of California, San Diego, La Jolla, CA, USA; 2 Department of Neuroscience, Columbia University, New York, NY, USA; 3 Mortimer B. Zuckerman Mind Brain Behavior Institute, Columbia University, New York, NY, USA; 4 The Kavli Institute for Brain Science, Columbia University, New York, NY, USA; 5 Halıcıoğlu Data Science Institute, University of California, San Diego, La Jolla, CA, USA; 6 These authors contributed equally; 7 Lead contact

## Abstract

The hippocampus plays a critical role in spatial navigation and episodic memory. However, research on *in vivo* hippocampal activity dynamics mostly relies on single modalities, such as electrical recordings or optical imaging, with respectively limited spatial and temporal resolution. Here, we develop the E-Cannula, integrating fully transparent graphene microelectrodes with imaging cannula, which enables simultaneous electrical recording and two-photon calcium imaging from the exact same neural populations across an anatomically extended region of the mouse hippocampal CA1 stably across several days. The large-scale multimodal recordings show that sharp wave ripples (SWRs) exhibit spatiotemporal wave patterns along multiple axes in two-dimensional (2D) space with different spatial extents and temporal propagation modes. Notably, distinct SWR wave patterns are associated with the selective recruitment of orthogonal CA1 cell assemblies. These results demonstrate the utility of the E-Cannula as a versatile neurotechnology with the potential for future integration with other optical components.

## In brief

Liu et al. develop the E-Cannula, integrating fully transparent microelectrodes with imaging cannula for reliable, long-term monitoring of cellular calcium response, local field potentials, and spiking activities from the same neuronal population.

## Graphical Abstract

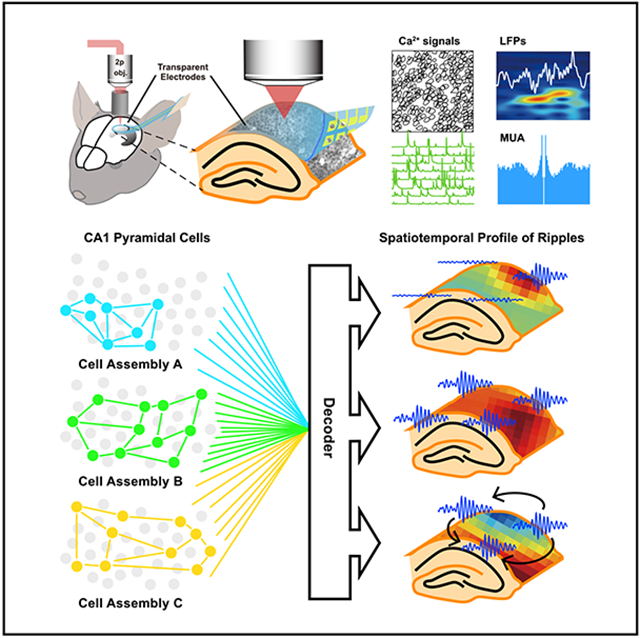

## INTRODUCTION

Large-scale electrophysiological and optical recordings have significantly advanced our understanding of neural circuit dynamics in the mammalian neocortex. However, similar experiments and analyses remain challenging in circuits that are located deep in the brain, such as the hippocampus, a region critical for spatial navigation and episodic memory (Buzsáki and Moser, 2013; Squire and Wixted, 2011). To date, most of the knowledge about hippocampal neural dynamics comes from experimental studies tracking responses from a small number of neurons monitored by conventional microelectrodes. However, understanding hippocampal functions requires monitoring the activity of large populations of hippocampal cells while recording the network level oscillations that both drive and are driven by their activity. Optical imaging techniques are optimal for monitoring cellular responses from large neuronal populations, but they do not provide the temporal resolution required for the simultaneous detection and characterization of network oscillations. The technological gap in directly monitoring large numbers of identified neuronal types and simultaneously recording fast time scale responses generated by hippocampal networks remains a major outstanding challenge. In response to this unmet need, we have developed the “E-Cannula” technology, a two-photon imaging window cannula integrated with transparent graphene microelectrodes for simultaneous optical imaging and electrical recordings from the same neural populations across large areas.

Hippocampal sharp wave ripples (SWRs) are the most synchronous population pattern recorded in the mammalian brain (Buzsáki, 2015). They have been hypothesized to control the information transfer from the hippocampus to down-stream cortical structures for learning and memory consolidation as well as planning, credit assignment, and prediction (Buzsáki, 2015; Girardeau et al., 2009; Jadhav et al., 2012; Ambrose et al., 2016; Carr et al., 2011; Dupret et al., 2010; Fernández-Ruiz et al., 2019; Pfeiffer and Foster, 2013). In this work, we applied the E-Cannula to obtain a dynamic map of SWRs in the CA1 region of the mouse hippocampus through simultaneous two-photon calcium imaging and direct recordings of SWR generation and propagation across the same population of cells. To date, *in vivo* studies into SWR-associated hippocampal neuronal dynamics have almost exclusively utilized electrophysiological recordings with single- or multiple-shank penetrating probes, with only a few instances of two-photon calcium imaging with simultaneous contralateral local field potential recordings (Grosmark et al., 2021; Malvache et al., 2016). Despite advances in imaging technologies, the relationship between (1) the micro- and mesoscopic patterns of population recruitment to SWRs accessible through high-resolution imaging methods and (2) the observed variability in the macroscopic structure of SWRs along large extents of the hippocampal septo-temporal and proximodistal axes accessible through large-scale hippocampal surface recordings remain unexamined. Therefore, it still remains unknown how cell recruitment by SWRs could be coordinated by topographic diversity in SWR events. To address these questions, we implanted the E-Cannula above the hippocampal area CA1 and performed simultaneous electrophysiological recording and two-photon calcium imaging of the intact CA1 region in head-fixed mice. We recorded both the multiunit activity (MUA) and SWRs while simultaneously monitoring fluorescence calcium signals from CA1 pyramidal neurons. We found that the MUA activity recorded by transparent microelectrodes increased during SWR events and was strongly phase locked to nearby SWR events. SWRs often co-occurred with high synchrony events (HSEs) detected with imaging, which involved co-activation of multiple neurons. We investigated the SWR activities recorded with transparent microelectrodes and found that SWR activity could be spatially local or global across the CA1 region and could also exhibit stationary or traveling temporal characteristics. By performing decoding analysis, we found that the SWRs with different spatiotemporal patterns were associated with distinct patterns of underlying hippocampal neural activity. Finally, most hippocampal cells formed orthogonal cell assemblies whose activation was selective to SWRs displaying specific electric spatiotemporal patterns.

## RESULTS

### *In vivo* multimodal recordings from hippocampal area CA1 with E-Cannula

**Figure 1.**
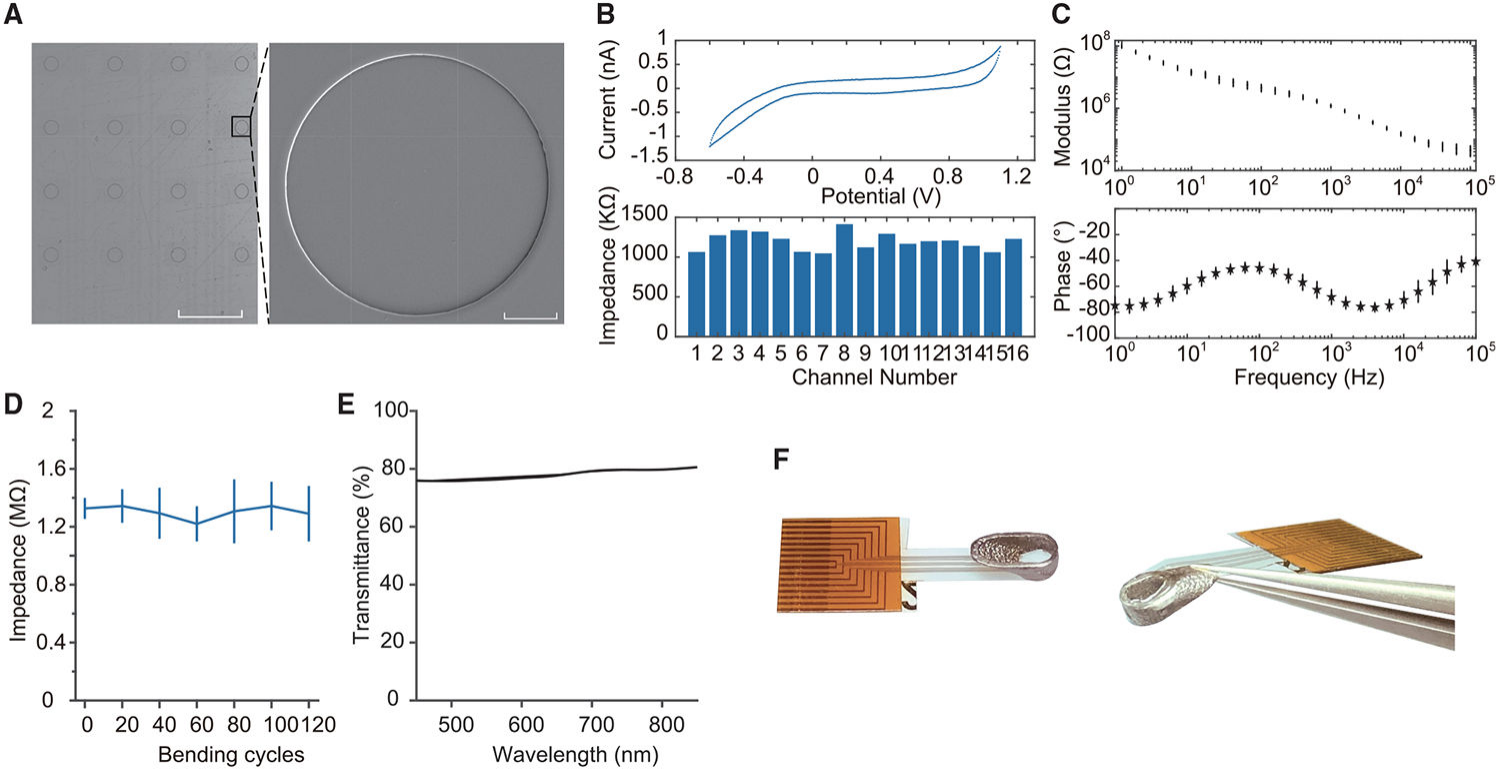
Fully transparent graphene microelectrode array and the integrated E-Cannula (A) Microscope image of the fully transparent 16-channel graphene microelectrode array (left) and the SEM image of one electrode (right). Left scale bar: 500 μm. Right scale bar: 20 μm. (B) The cyclic voltammetry result of one example electrode (top) and the impedance of 16 microelectrodes (bottom). (C) The electrochemical impedance spectroscopy result showing the magnitude (top) and phase (bottom) of one example electrode. (D) The result of bending test for the graphene array, showing stable impedance after at least 120 bending cycles. The line is mean, and the error bars indicate STD. (E) Transmittance of the graphene array under different wavelengths. (F) Picture of the fully transparent array, the imaging cannula, and the integrated E-Cannula in top view (left) and side view (right).

In order to construct E-Cannula, we fabricated fully transparent graphene microelectrodes on flexible and transparent polyethylene terephthalate (PET) substrate (Figure 1A). We employed an electrochemical delamination graphene transfer method and a previously developed 4-step cleaning method (Thunemann et al., 2018) for the fabrication (see STAR Methods for details on the fabrication process). The transparent array consists of 16,100 μm diameter circular electrodes with 500 μm spacing. The scanning electron microscopic image shows the profile of the well-defined electrode openings (Figure 1A). The total area of the array was 2.3 × 1.8 mm to match the opening of the imaging cannula. The small size of the array was key to minimizing damage during implantation surgery. In order to reduce the overall array size without sacrificing the number of recording channels, we replaced the previous gold connecting wires with double-layer graphene wires and carefully routed the wires through the inner plane of the array instead of surrounding the array, significantly reducing the total size of the array by ~4× times compared with the previous configuration (Thunemann et al., 2018). We performed cyclic voltammetry and electrochemical impedance spectroscopy to characterize the graphene microelectrodes in 0.01 M phosphate-buffered saline (Figures 1B and 1C). The result from cyclic voltammetry measurements shows no redox peaks, suggesting that the electrode-electrolyte interface is dominated by the double-layer capacitance (Figure 1B). Our electrodes exhibited a uniform impedance across channels with an average value of 1.1 MΩ at 1 kHz, achieving 100% yield (Figure 1B).

The flexibility and sturdiness of the transparent microelectrode arrays proved critical for their integration with the imaging cannula and their continued durability during chronic *in vivo* experiments. We characterized the reliability of the array with bending tests, where the array was bent to a radius of 5 mm. The results show that the impedance of the array stays stable even after 120 bending cycles (Figure 1D), demonstrating the high reliability of the array suitable for integration with the imaging cannula used in this study. Besides that, the array also exhibits high transparency across the wavelength ranges commonly used for optical imaging (Figure 1E). Figure 1F shows a picture of the fully transparent graphene microelectrode array, the imaging cannula, and the assembled E-Cannula. The flexible shank of the graphene microelectrode array was bent and attached to the outer wall of the imaging cannula and the fully transparent microelectrode array provided a completely clear FOV. In the present configuration, the cannula was designed to have a ~30° angle. In practice, we found that the electrode impedance of the graphene array stayed roughly unchanged even after 90° bending (Figures S1A and S1B), making it compatible with cannulas of 90° angle, thus further facilitating implantation.

**Figure 2.**
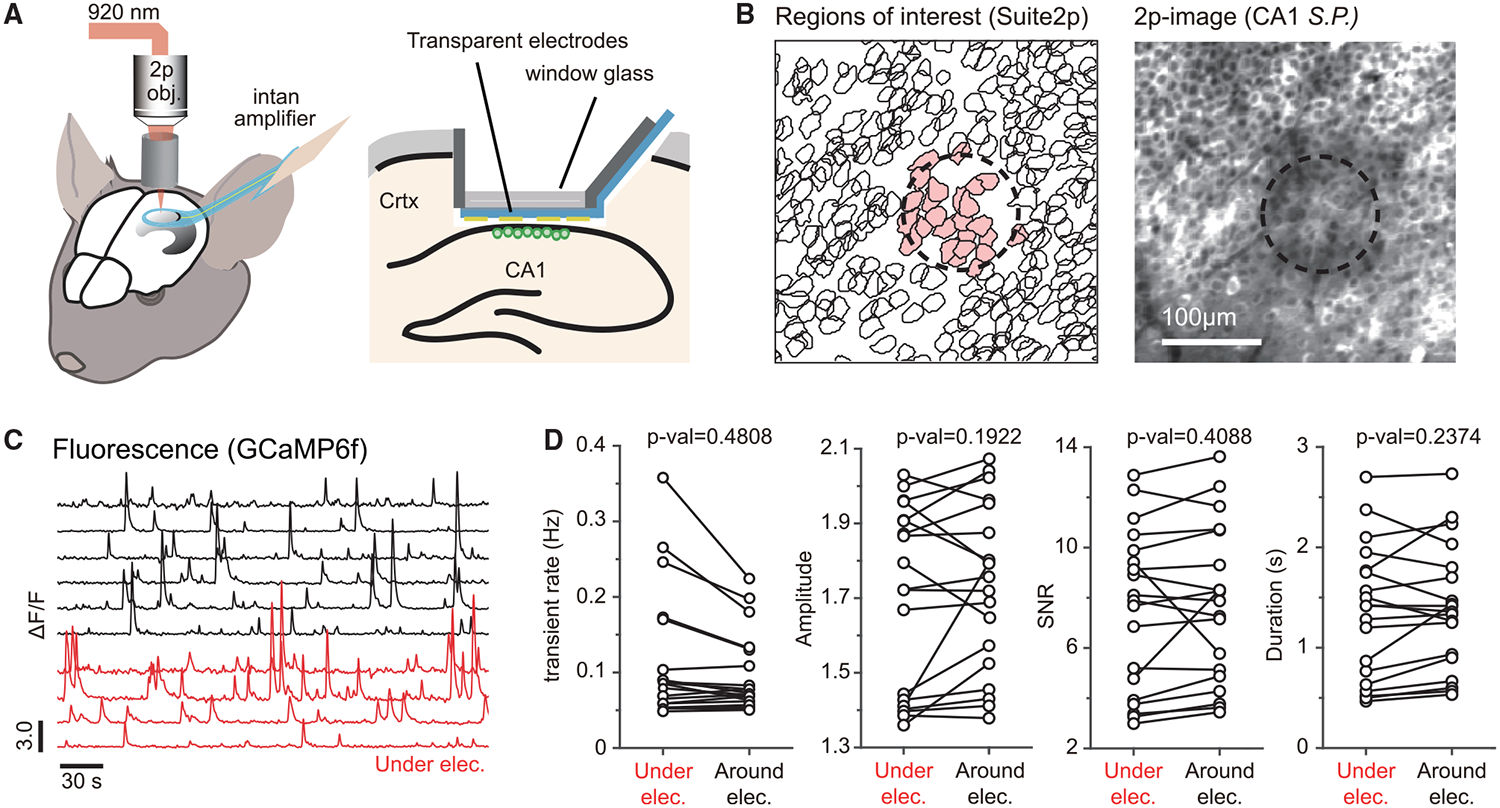
Simultaneous two-photon imaging and electrical recording from hippocampal CA1 area in awake mice (A) Schematics of *in vivo* multimodal recording from the dorsal CA1. The transparent graphene microelectrode array was attached to the bottom of the cannula and placed on the alvear surface of the hippocampus. (B) Example small area around a recording electrode within a larger imaging field of view (840 × 840 μm^2^) with imaging plane in the CA1 stratum pyramidale (S.P.). The dashed circle shows the location of the electrode. Scale bar: 100 μm. Left: suite2p-detected regions of interest (ROIs) of CA1 pyramidal cells under (red shaded) and around the electrode. Right: time-averaged two-photon image (wild-type [WT] mouse injected with CamKII promoter virus). (C) Representative GCaMP calcium signals (ΔF/F0) extracted from cell ROIs around (black, n = 6) and under (red, n = 4) the recording electrode. (D) Comparison of the transient rate, amplitude, SNR, and duration of GCaMP-calcium events between cells directly under the graphene electrode and around the graphene electrode, showing similar activity properties. Each dot represents the median value from cell activities in one recording session. The observed cell population in each recording session is different. Two-tailed bootstrap test (10,000 times, n = 19 recording sessions from 3 mice).

With the fully transparent graphene microelectrode arrays, we performed simultaneous two-photon GCaMP-calcium imaging and electrophysiological recordings from the same hemisphere of CA1 (Figure 2A; see STAR Methods), while mice ran voluntarily and rested on a circular treadmill (Danielson et al., 2016; Zaremba et al., 2017). The E-Cannula was placed over the CA1 alveus of the hippocampus after aspiration of the underlying cortical tissues (see STAR Methods), and 840 × 840 μm^2^ images, 4 electrodes of the array that are typically covered, were acquired at 30 Hz from CA1 stratum pyramidale. As shown in Figures 2B and 2C, large populations of CA1 pyramidal cells were successfully imaged with single-cell resolution through the transparent graphene array (1,079 ± 43, mean ± SEM cells per field of view [FOV]) and stably identified over 20 days after viral injections (Figures S1C-S1F).

To confirm that the electrode attachment to cannula glass does not degrade the imaging quality, we compared the calcium signals imaged through E-cannula and standard cannula. Both signal properties are similar in signal-to-noise ratio (SNR), transient amplitude, and duration (Figure S2). We also characterized the fluorescence activity between the cells under the graphene electrode versus around the graphene electrode and found similar amplitude, duration, event rate, and SNR of calcium events (Figure 2D). These results confirm that our E-cannula provides similar imaging quality compared with standard imaging cannula. Furthermore, unlike conventional metal-based electrode arrays, transparent graphene microelectrodes do not generate any light-induced artifacts that would otherwise interfere with electrophysiological recordings (Thunemann et al., 2018; Liu et al., 2018). Photo-induced currents are intrinsically very weak and fast in graphene, requiring special structures or extremely low temperatures to even detect them (Lemme et al., 2011; Gabor et al., 2011).

We next examined features of local field potential (LFP) recorded from the array. We observed clear theta (4–8 Hz) oscillations in the recordings when the animal was running (Figures S3A and S3B). Consistent with the previous reports (Tort et al., 2008), we also observed phase-amplitude coupling in delta-gamma and theta-gamma bands (Figure S3C), indicating that CA1 stratum pyramidale and stratum oriens were the main sources for the recorded LFPs. In a previous study, theta waves were reported to travel along the septotemporal axis of the hippocampus (Patel et al., 2012; Lubenov and Siapas, 2009). In our data, we also observed that the theta oscillations traveled along the septotemporal axis in the two-dimensional (2D) recording space with a speed of 0.1484 m/s (Figures S3D and S3E), which is similar to the results reported in the previous study (Patel et al., 2012).

**Figure 3.**
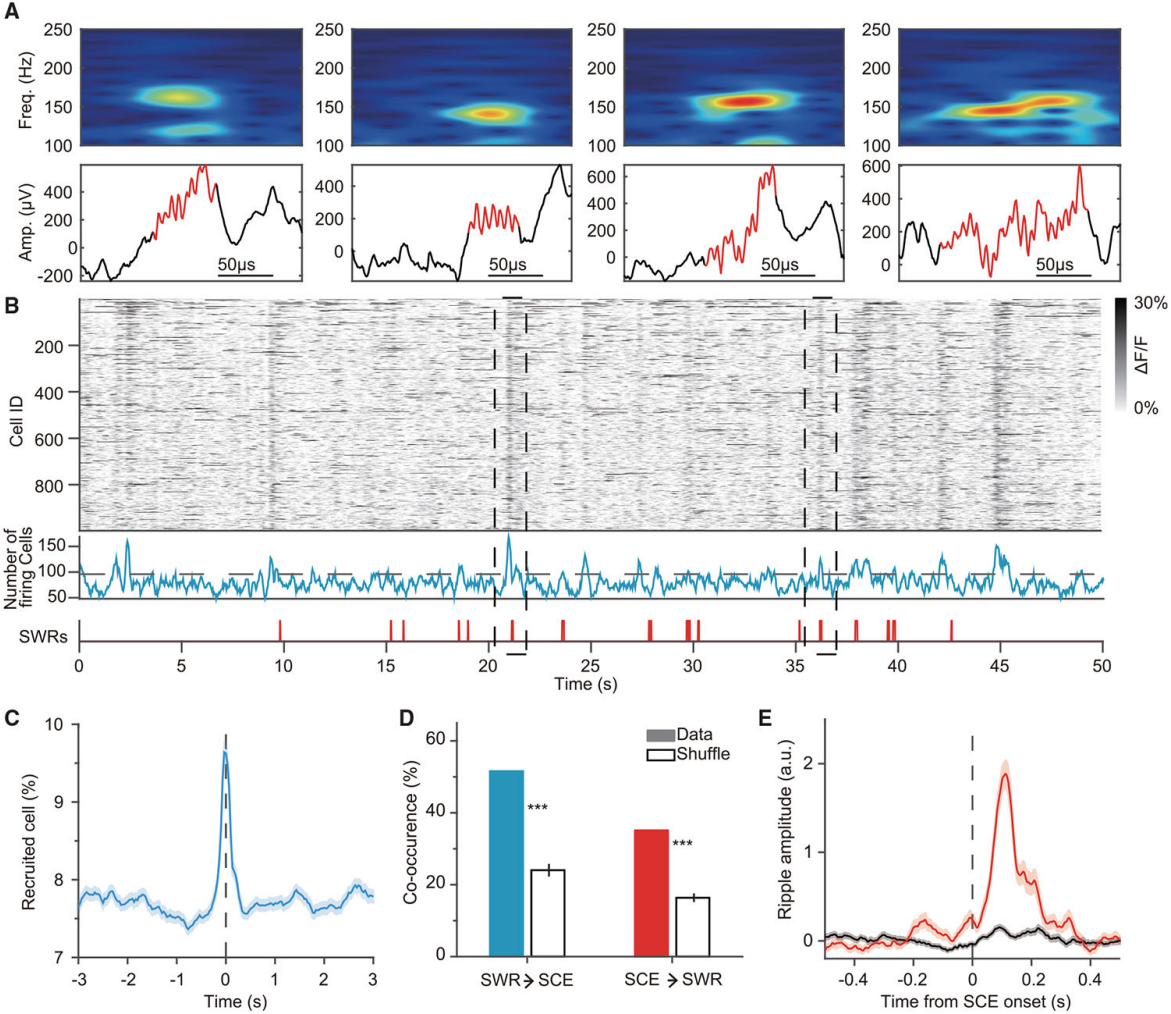
Synchronous dynamics of CA1 pyramidal cells during SWRs (A) Examples of ripples detected on the electrode (top: ripple band power spectrogram, 100–250 Hz; bottom: LFP traces). (B) SWR-associated SCE of CA1 pyramidal cells. ΔF/F of the cells (n = 998) during immobile states (top), and the number of the active cells over time frames (middle). Black dashed line indicates significance threshold for SCE detection. The onsets of detected SWRs on the electrode above the FOV (bottom). Black dashed lines indicate co-occurrence events of SCEs and SWRs. (C) Peri-stimulus time histogram of percentage of firing pyramidal cells around local SWR onsets (n = 570) from a session. Shaded region indicates SEM. (D) The co-occurrence rate between SWRs (n = 570) and SCEs (n = 249) from the same session used in (C) (two-tailed shuffling test, 10,000 times). The error bars indicate the SEM. ***p < 0.001. SWR→SCE: data 51.58, shuffle 24.03 ± 1.76, mean ± SEM. SCE→SWR: data 35.13, shuffle 16.37 ± 1.2, mean ± SEM. (E) Peri-stimulus time histogram of *Z* scored SWR envelope detected by the electrode above the FOV around SCEs (red, n = 249) and non-SCEs (black, n = 588) from the same session used in (C). Shaded regions indicate SEM.

We then employed E-Cannula to record SWRs across a wide area of dorsal CA1. Representative waveforms and the power spectrograms of detected SWRs are shown in Figure 3A. In agreement with previous electrophysiological and optical recordings (Buzsáki, 2015; Grosmark et al., 2021; Malvache et al., 2016; Joo and Frank, 2018), we clearly observed that a large number of CA1 pyramidal cells were sharply activated during the SWRs detected in the channels within FOVs (Figures 3B and 3C). A significant fraction of synchronous calcium events (SCEs) co-occurred with SWRs (51% SWRs to SCEs, and 35% SCEs to SWRs; Figure 3D), and SCEs were associated with a robust increase in ripple band envelope power (Figure 3E). To compare with the previous reports using contralateral recording, we examined how SCEs are differently associated with ipsilateral and contralateral SWRs (Figure S3F). We observed that SCEs more often accompanied ipsilateral than contralateral SWRs (Figure S3G), corresponding to the moderate co-occurrence of SWR events between the hemispheres (39%; Figures S3H-S3K). Moreover, we found that the spatial profiles of ipsilateral SWRs correlated with the duration of contralateral SWRs but not the amplitudes (Figure S3L). Together, the E-Cannula design allows us to simultaneously monitor calcium dynamics in large populations of individually identified cells and to more precisely relate these to the prominent oscillatory electrical potentials directly recorded from imaging FOVs.

### Multiunit activities recorded by E-Cannula from hippocampus

**Figure 4.**
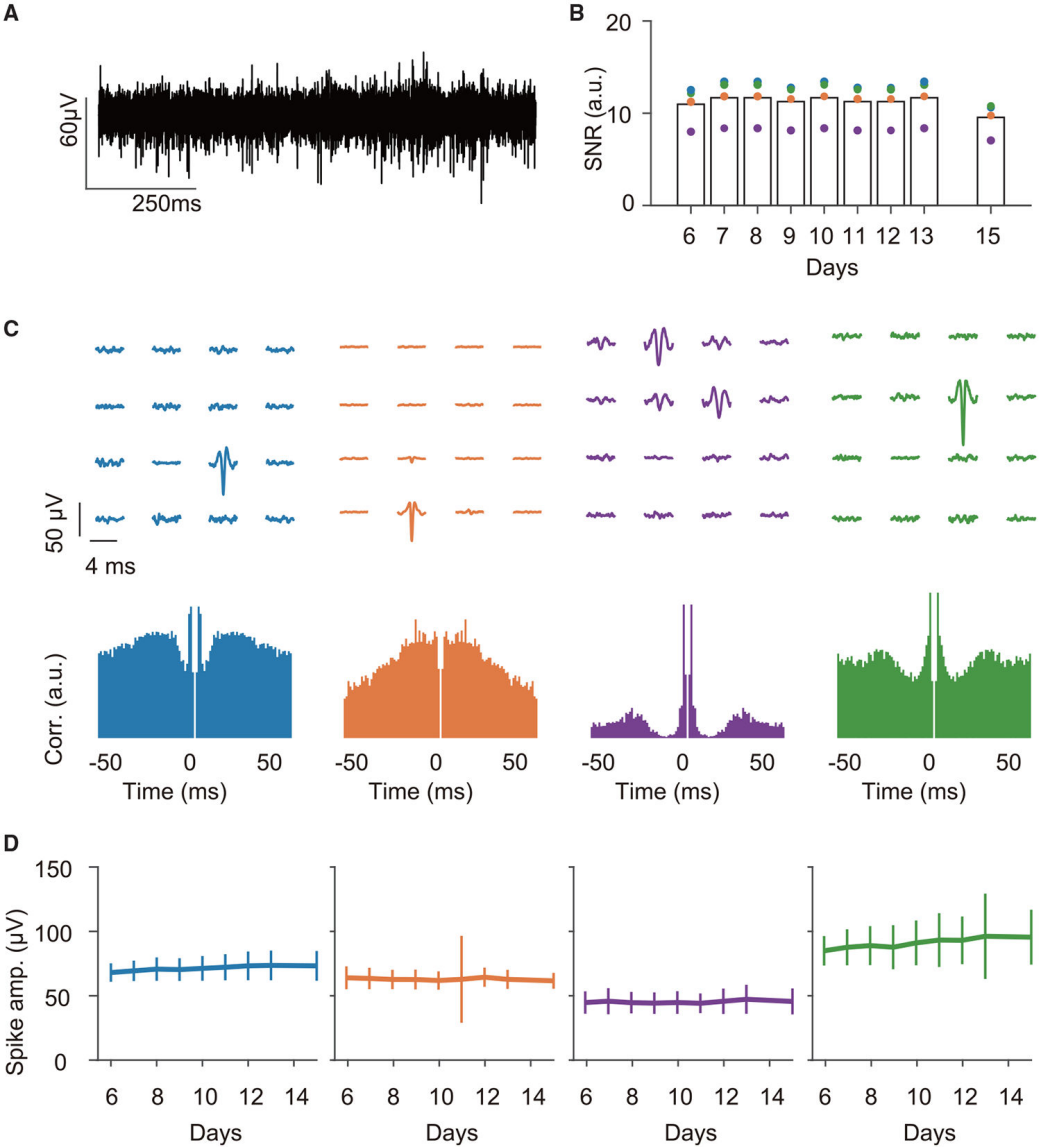
Hippocampal putative spikes recorded by the 2D graphene array (A) Representative LFP trace from one electrode after high-pass filtering at 500 Hz. (B) SNRs of the detected spikes from an animal at different days. Those detected spike clusters have stable SNRs across days. (C) Spatial profiles of the mean waveforms for each spike cluster (top) and the autocorrelograms (bottom). The spike waveforms were mainly detected on a single channel. The data are from the same animal as in (B). (D) Spike amplitude of each cluster at different days after implantation, showing a stable recording for putative spikes across days. The data are from the same animal as in (B). The lines are means, and the error bars indicate STD.

**Figure 5.**
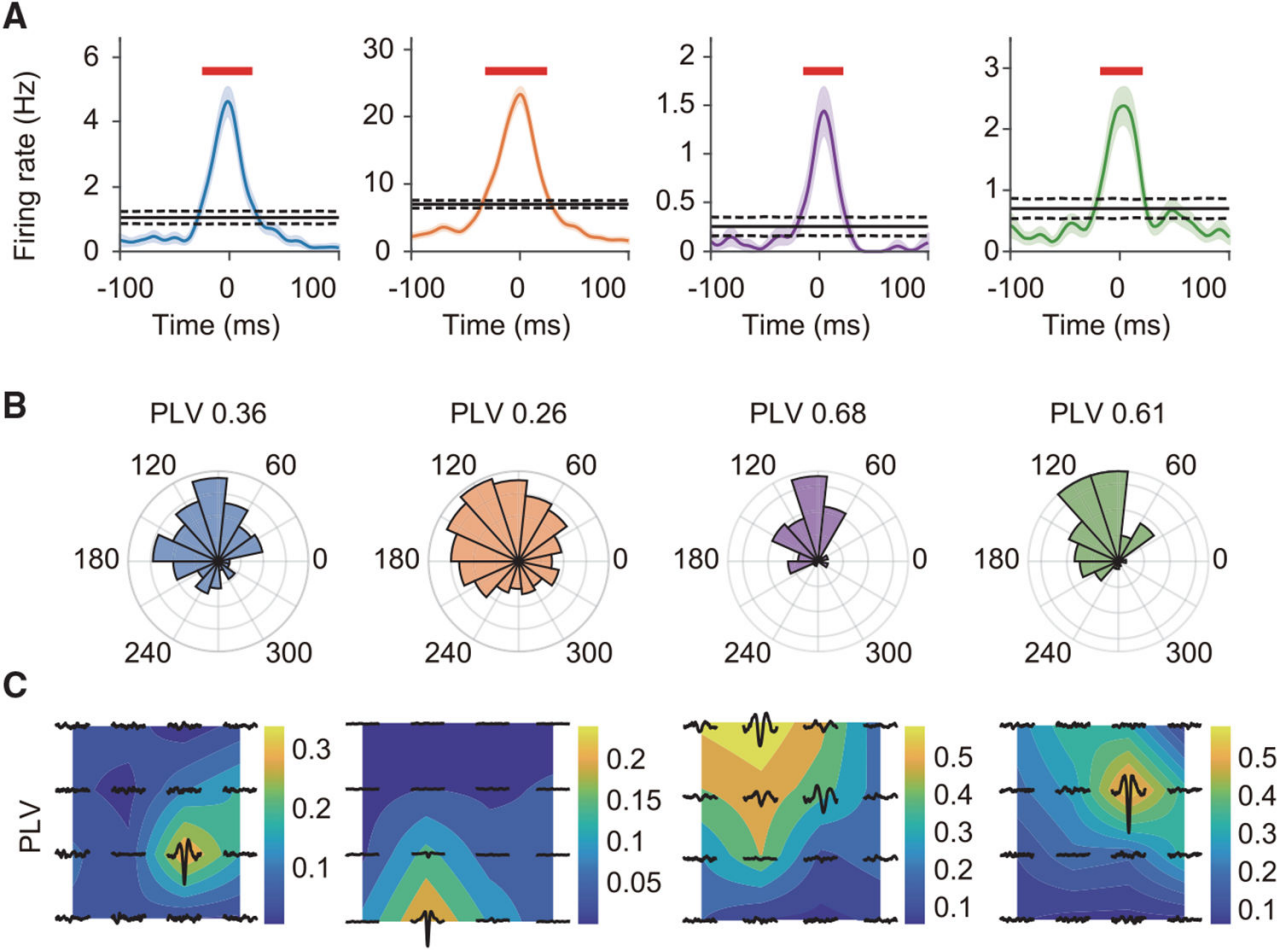
Locally controlled phase modulation of MUA spikes by SWRs (A) Firing rates of each spike cluster shown in Figure 4 during SWR events (n = 1,233) detected on the same channel in one recording session. All the spike clusters increase their firing rates around the SWR onset. Shaded regions indicate SEM. (B) The ripple band phase distribution at the putative spike firing times from the same session as in (A). All the spikes are phase locked to the falling edge of the ripple waveforms. (C) The phase-locking values (PLVs) between the firing times of each spike cluster and the phase of SWRs at different electrodes. The putative spikes were mainly phase locked to the SWR signals from the adjacent electrodes. The data are from the same session as in (A).

Since previous studies have demonstrated that the spiking activity of hippocampal neurons could be detected at distance of up to 150–200 μm (Harris et al., 2000; Buzsáki and Kandel, 1998; Khodagholy et al., 2015), we examined whether our transparent graphene microelectrodes could also detect spiking activity from the hippocampus. Figure 4A shows an example signal after high-pass filtering at 500 Hz. Using Kilosort (see STAR Methods), spikes were detected from multiple channels and sorted into discrete MUA clusters (number of clusters per mouse: 2.25 ± 0.75, firing rate: 0.77 ± 0.32 Hz, mean ± SEM) that exhibited stable SNRs across multiple days after implantation (Figure 4B). The spikes of each MUA were typically detected in one of the channels (Figure 4C) and stably exhibited across multiple days (Figure 4D). Detected spike waveforms showed both symmetric and asymmetric shapes, with most MUA clusters exhibiting a low firing rate (Figure S4A), suggesting that the detected MUA clusters primarily originated from pyramidal cells (Csicsvari et al., 1999; Skaggs et al., 1996). We further sought to determine whether spike timing of MUA clusters was modulated by SWRs. Consistent with previous findings that SWRs are associated with a robust increase in CA1 neuronal firing rate (Csicsvari et al., 2000), we found that all the clusters clearly increased their firing rates around the onset of the SWR events detected on the same channel (Figure 5A). The MUA spikes were phase locked to SWRs, predominantly occurring at the rising edge of the ripple oscillation (Figure 5B), while the ripple-frequency phase modulation of MUA gradually attenuated as the distance to the ripple channel increased (Figure 5C). Therefore, MUA clusters detected in the graphene array mainly included action potentials from neurons proximal to each channel, and phase modulation by SWRs could operate at local circuit level. Finally, we checked the simultaneously imaged calcium ΔF/F signals from the cells right below one recording channel and compared them with spikes detected from the same channel (Figure S4B). We first identified the calcium transient events of the cells around the electrode (100 μm to the electrode center) that detect the MUA activity. Then, we computed the number of overlapping MUA spikes during calcium transients for each cell and compared it with the number of overlapping MUA spikes after shuffling the MUA spike timing 10,000 times. We computed the p value and used Benjamini-Hochberg false discovery rate (FDR) correction for multiple comparisons. The results indeed show significant matching between the MUA spikes and a few cells around the same electrode (Figure S4B). Together, these results demonstrate that E-Cannula facilitates simultaneous detection of electrical and optical signals from hippocampal cells.

### Spatiotemporal characteristics of SWR events and their correspondence with hippocampal neuronal activity

**Figure 6.**
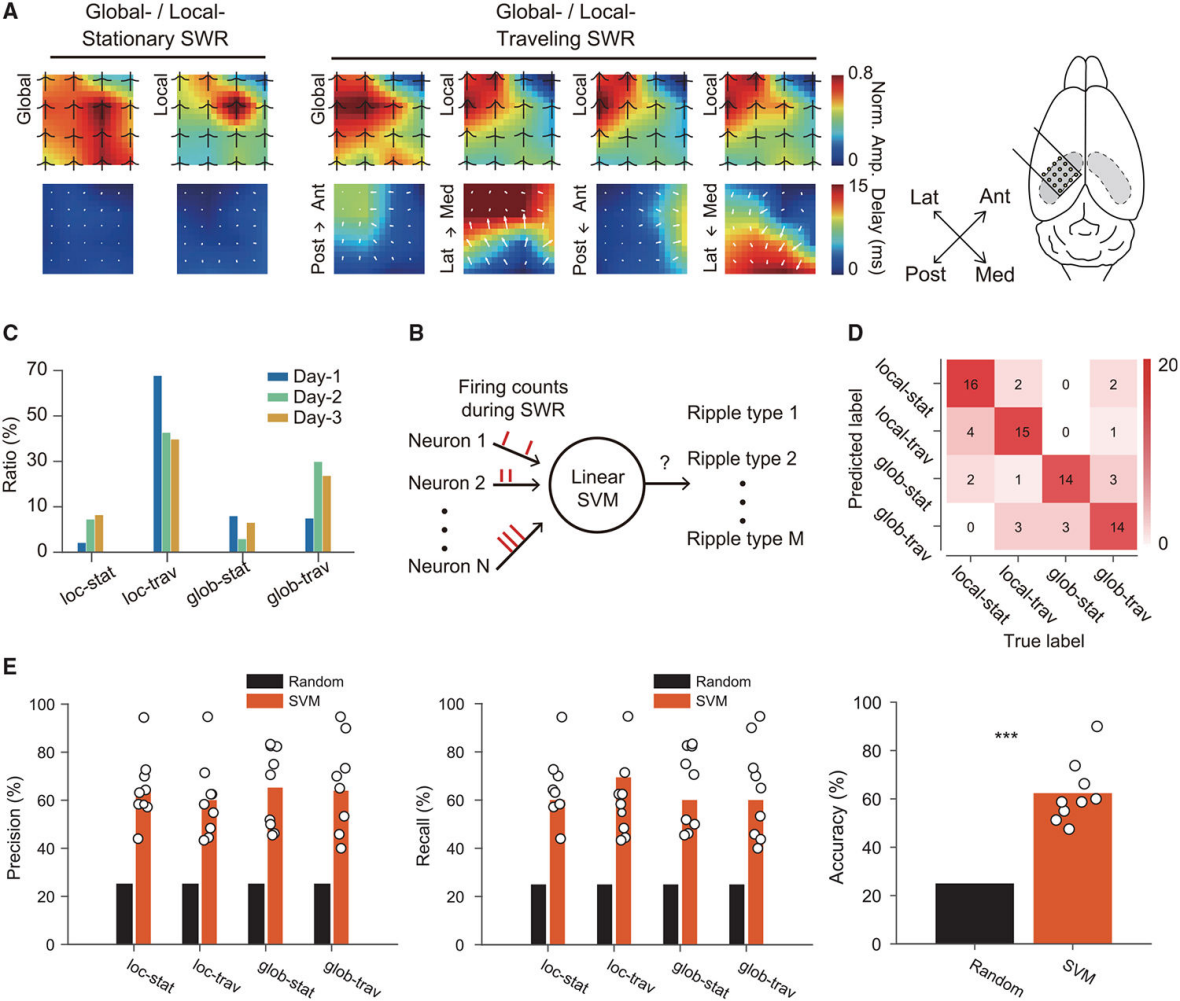
Spatiotemporal patterns of SWRs decoded by CA1 local circuit activity (A) Identified SWR clusters showing different spatial activation patterns (top) and temporal delays (bottom) relative to the septotemporal and transverse axes of the hippocampus. The black traces on the top row show the average ripple envelope detected at each channel. The white arrows on the bottom row show the numerical gradient (Med: medial; Lat: lateral; Ant: anterior; Post: posterior). (B) Ratio of SWR events with different spatiotemporal characteristics across recording days. (C) Decoding of SWR types based on cellular calcium signals around SWR onsets. (D) Confusion matrix for decoding the spatiotemporal characteristics of SWRs as a representative example from a session. (E) Precision, recall, and accuracy for decoding the spatiotemporal characteristics of SWRs from all three mice for days 1–3 combined (two-tailed bootstrap test, 10,000 times, ***p < 0.001, n = 9 sessions).

Given that SWRs are generated by transient neuronal discharges propagating across the hippocampal circuit (Csicsvari et al., 2000; Oliva et al., 2016; Stark et al., 2014), we hypothesized that SWRs could be biased to an anatomical extent along the septotemporal and transverse axes rather than being universally synchronized. To test this hypothesis, we first computed the power and peak latency of recorded SWRs for each channel relative to all the channels across the array. Then, we carried out K-means clustering to identify clusters with distinct spatiotemporal patterns (see STAR Methods). We found multiple SWR clusters characterized by different spatial occurrences and temporal propagation modes, which were further classified into four groups (local stationary, local traveling, global stationary, and global traveling) as shown in Figure 6A. The ratio of SWR events assigned to different clusters across days after implantation is shown in Figure 6B.

We next sought to characterize neuronal activity patterns associated with SWR clusters with different spatiotemporal characteristics along the septotemporal and transverse axes of the hippocampus. For this, we applied decoding analysis with support vector machines (SVMs) to ask whether the four groups of SWR events could be discriminated based on the neuronal activities (Figure 6C; see STAR Methods). We used the calcium signals of the imaged cells during SWRs as the input features and decoded the group identity of SWR events and performed recursive feature elimination to select the best subset of neurons that are most informative about the SWR cluster type. All the SWR clusters were successfully decoded with high accuracy, precision, and recall above chance levels (Figures 6D, 6E, and S4C). These results suggest that the spatiotemporal bias of SWRs is a potential mechanism for the selective activation of specific hippocampal cell assemblies.

### Differential recruitment of cell assemblies to SWR clusters

**Figure 7.**
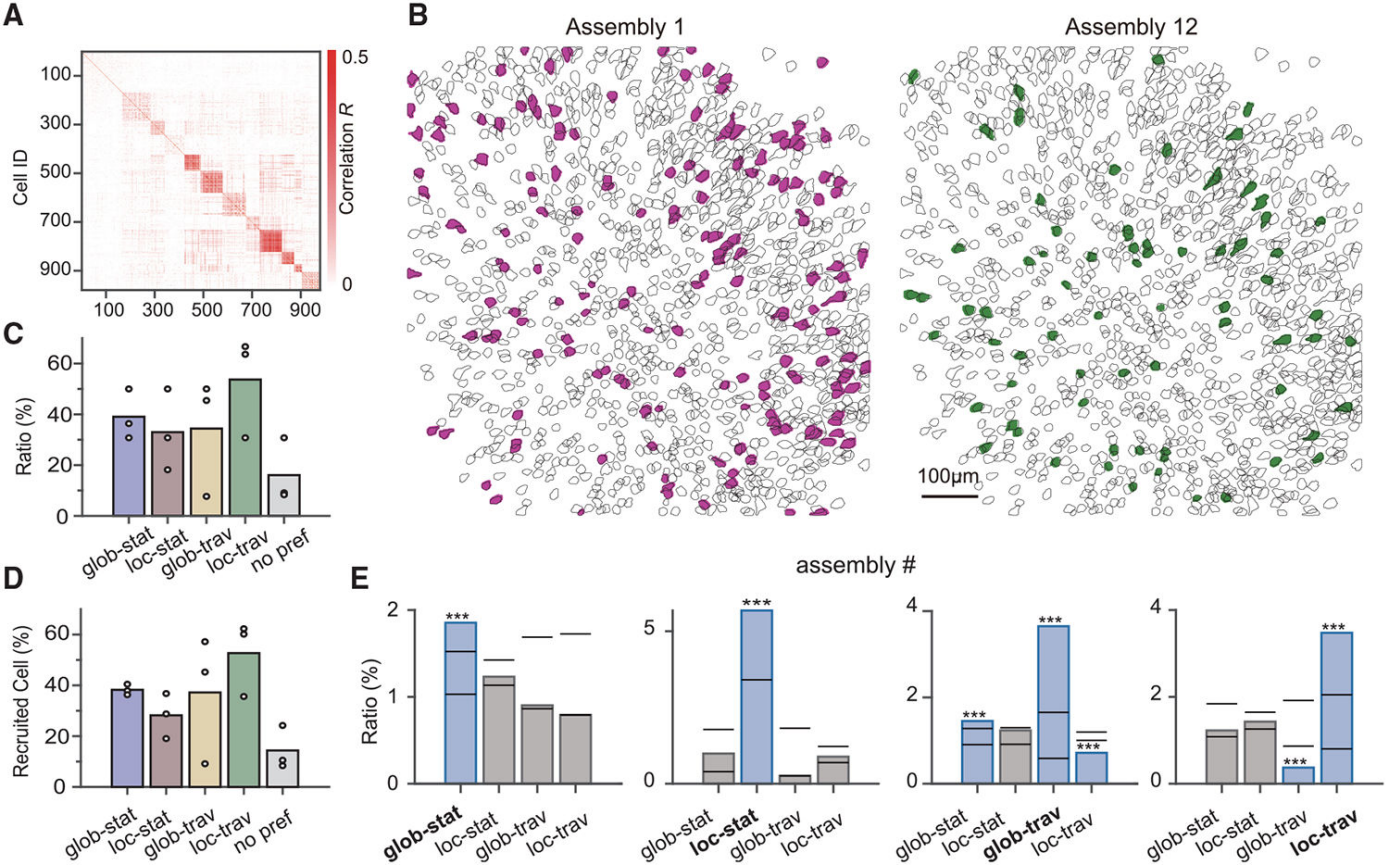
CA1 cell assemblies differently activated during spatiotemporal-profiled SWRs (A) Representative example of adjacency matrix of the identified cell assemblies (n = 979) from one mouse (WT mouse injected with CamKII promoter virus). Adjacency matrices for other mice are shown in Figure S6. (B) Representative examples of topological distribution of cells for two assemblies from the same mouse as in (A). The rest of the assemblies are shown in Figure S5. (C) Ratio of cell assemblies associated with SWR events of different spatiotemporal characteristics for all three mice. Each dot represents data from one animal. (D) Ratio of recruited cells from assemblies associated with SWR events of different spatiotemporal characteristics for all three mice. Each dot represents data from one animal. (E) The firing ratio of four representative cell assemblies under different SWR clusters. All cell assemblies show significant higher/lower participation ratios under different clusters. The ratio of each cell assembly under each SWR cluster was tested by shuffling test (n = 10,000 times, p < 0.05 with FDR correction, *p < 0.05, **p < 0.01, ***p < 0.001). The bars are the 2.5 percentile and 97.5 percentile values obtained from shuffled data. The firing ratios for all cell assemblies for all mice are shown in Figure S6.

Having seen that the SWR cluster identity could be decoded from the calcium activity dynamics of hippocampal neuronal populations, we asked whether individual neurons formed into cell assemblies that exhibited segregated activities under different SWR clusters. For this, we computed the similarity between the calcium activity of recruited cells during each SWR event and performed clustering with community detection to assign the cells into different groups (see STAR Methods). We identified multiple cell assemblies with sizes ranging from 30 to 140 cells (Figure 7A); the topological distribution of the cell assemblies was unbiased with respect to the anatomical coordinates of cells within the FOVs (Figures 7B and S5). To examine the associations between each cell assembly and the SWR clusters, we analyzed the firing ratio of the cell assemblies during each SWR cluster. Notably, we found that most of the cell assemblies exhibited selective firing that was higher or lower than average for specific SWR clusters and that the preferred cluster identity varied across assemblies (Figures 7C-7E and S6). As in the previous report (Malvache et al., 2016), we observed that several assemblies were activated together at several ripple events (8.5%; Figures S7A and S7B). While the number of activated cells is independent for the ripple amplitudes (Figure S7C), ripples with global or traveling profiles recruited more cells than local and stationary ones (Figure S7D). Finally, we addressed whether the assembly relationship is associated with the spatiotemporal bias of ripples using two different control analyses. First, we shuffled the assembly labels for all neurons (n = 1,000 times) and compared the decoding performance with the original labels (Figure S7E). To rule out the effect of spatial dispersion on decoding performance, we compared the average pairwise distance between neurons in cell populations used for these control analyses (Figure S7F). For the second control analysis, we compared the decoding performance using cells randomly selected from all assemblies (labeled as holding) and cells sequentially selected from a subset of assemblies (labeled as in order) (see STAR Methods). The decoding accuracy of spatiotemporal profiles of ripples was significantly deteriorated when the activities of a subset of cell assemblies were used as input features (Figure S7G). The spatial dispersion looked similar for both holding and in-order groups (Figure S7H). These results demonstrate that E-Cannula recordings allow for the identification of cell assemblies that are differentially recruited to SWR clusters with distinct spatiotemporal profiles along the major axes of the hippocampal CA1.

## DISCUSSION

In this study, we report the development and implementation of E-Cannula technology for simultaneous *in vivo* electrophysiological recordings and two-photon calcium imaging from the same hippocampal tissue volume. *In vivo* recordings of the hippocampal dynamics have mainly utilized technologies with single modality. With our E-Cannula, we demonstrate reliable multimodal recordings of MUA spikes, SWRs, and cellular calcium activity dynamics combined simultaneously in the same experiment. This combination of multiple modalities allowed us to carry out a comprehensive characterization of SWRs across spatial and temporal resolutions, providing a more detailed and complete picture of SWR-related spatiotemporal neural dynamics that was inaccessible for conventional single-modality approaches.

We find that the SWR activity across 2D space exhibits diverse spatiotemporal patterns, including different propagation directions and spatial spread. These results are consistent with previous studies using a penetrating electrode array with multiple shanks spanning in the 1D direction (Patel et al., 2013). Due to limitations on implanted electrode arrays, these reports were able to observe traveling SWR activities only along the anteroposterior axis. In our experiment, we confirmed the traveling behavior of SWR activities along this main axis but further report that SWRs exhibit variable spatial extent along multiple axes in 2D space.

While E-Cannula allows us to detect MUAs, the MUA firing rates are low. For the low firing rate of MUA, we suspect that each MUA cluster may only consist of a few low firing rate pyramidal cells. Also, considering the relatively high impedance of our E-Cannula (1.2 ± 0.1 Mohm, mean ± SEM) and the distance between the planar electrodes and the pyramidal cell layer, mainly large amplitude spikes, like complex spikes of pyramidal cells, were detectable. Despite these limitations, we indeed observe correspondence between the MUA spikes and the calcium activity of a few neurons under the recording channel. The ability of our approach to exactly register putative spikes recorded by the transparent electrodes to individual cells imaged directly beneath the electrode could be constrained by various factors. First, the calcium response may not reflect all of the spiking events of the cell, leading to potential false negative responses. Huang et al. (2021) showed that the cellular fluorescence activity could be undetectable when the cell’s firing activity is sparse or when cells are imaged at low magnification. Second, considering the electrode size and the distance to the pyramidal layer and the stratum oriens of the CA1 region, the detected MUA spikes likely originate from multiple pyramidal cells and potentially also from interneurons, further limiting reliable mapping of the detected spikes to imaging signals.

Recent studies have successfully combined LFP recordings from the hippocampus from one hemisphere with simultaneous two-photon calcium imaging from the other hemisphere (Grosmark et al., 2021; Malvache et al., 2016). They reported that the SCEs in the hippocampus co-occurred with the SWR events detected in the contralateral hippocampus. Our E-Cannula technology allowed simultaneous recordings of LFP and fluorescence activity from the same hippocampal neuron population. In our data, we observed a higher co-occurrence ratio between the SCEs and SWRs, revealing a more direct correspondence between the optically detected population events and the SWRs. Besides the co-occurrence ratio, it was reported that different cell assemblies were recruited during SCEs (Malvache et al., 2016). Also, a previous *in vitro* study reported that different SWR waveforms in a single recording channel could relate to underlying cellular activity patterns (Reichinnek et al., 2010). Our E-Cannula approach was able to replicate these findings and further demonstrate that these cell assemblies are differently recruited during SWR clusters with distinct spatiotemporal characteristics. Notably, the recruitment of unique cell assemblies by SWRs with distinct spatiotemporal patterns provides a potential mechanism for the orthogonal reactivation of assemblies associated with discrete memories while minimizing memory interference.

Besides graphene, other transparent electrode materials have been proposed in the literature (Kwon et al., 2013; Cho et al., 2022; Neto et al., 2021; Qiang et al., 2018; Liu et al., 2021). Indium tin oxide (ITO) has also been used for transparent microelectrode arrays (Kwon et al., 2013). However, compared with graphene, it is more brittle and susceptible to cracking and other mechanical degradations. PEDOT-PSS has been recently adopted to fabricate transparent electrodes (Cho et al., 2022), but this material system suffers from poor chronic reliability due to delamination issues. Silver nanowires (Neto et al., 2021) or gold meshes (Qiang et al., 2018) have been used to fabricate transparent electrodes, but these metals still absorb the light leading to light-induced artifacts. In addition, silver has been known to be toxic to the tissue. In summary, none of the above materials offer transparency, flexibility, long-term reliability, artifact-free recording capability, and biocompatibility simultaneously. Recently, our group has developed a penetrating probe technology, Neuro-FITM, to perform multimodal recordings combining wide-field imaging with electrophysiological recordings from subcortical structures such as the hippocampus (Liu et al., 2021). High transparency of the shank of Neuro-FITM allows wide-field imaging without creating shadows or blocking the FOV. However, it should be noted that the tip of Neuro-FITM probes is made of thin gold wires and platinum electrodes, which are not transparent. Due to its laminar structure and non-transparent microelectrode, Neuro-FITM would not be suitable for integrating with regular imaging cannula to perform two-photon imaging and recording from the same region of the hippocampus.

Our study demonstrated a successful combination of simultaneous 2D electrical recordings and two-photon calcium imaging from a large area in the hippocampus. In the future, the spatial resolution of electrical recordings by E-cannula could be further enhanced by reducing both the size of the graphene electrode and the spacing between adjacent recording channels, enabling the investigation of hippocampal CA1 neural circuits at a finer scale. Also, shrinking the electrode size and pitch will improve the spike recording precision and electrical source separation of the E-cannula, making it possible to simultaneously record calcium signals and action potentials from the same neurons *in vivo*. Future work could further leverage the temporal resolution of E-cannula electrical recordings with the cellular and genetic targeting techniques utilized in calcium and voltage imaging to dissect the interaction between the activity of specific cellular motifs and anatomically heterogeneous network oscillations. Moreover, the demonstrated electrical recording and imaging stability of the E-cannula is ideally suited for future comprehensive longitudinal tracking of the cellular and regional network changes underlying long-term memory. Finally, the above-mentioned advantages of the E-cannula system make it well suited for advancing research on the mechanistic relationships between local and global markers of activity across time. This research, in turn, may have implications in the development of robust brain-machine interfaces. Therefore, our E-Cannula approach and associated experimental protocols have the potential to advance the scope of future chronic recording experiments, including facilitating the dissection of the large-scale hippocampal activity dynamics that underlie learning and memory consolidation.

### Limitations of the study

The current electrode has an opening size of 100 μm and spacing of 500 μm, which impedes our investigations of matching between the electrical spikes and cellular fluorescence activity. Further technical improvements are needed to reduce the spatial scales of the electrode array to enhance the spike detection capability of E-Cannula. Although we demonstrated the association between cell assembly activities and spatiotemporal characteristics of SWRs, further investigations are needed to find out how this association developed during memory consolidation.

## STAR★METHODS

### RESOURCE AVAILABILITY

#### Lead contact

Further information and requests for resources should be directed to and will be fulfilled by the Lead Contact, Duygu Kuzum (dkuzum@eng.ucsd.edu).

#### Materials availability

This study did not generate new unique reagents.

#### Data and code availability

- The data for this study is available upon request from the lead contact.
- All original code has been deposited at Zenodo (https://zenodo.org/record/7055600) and is publicly available as of the date of publication. DOIs are listed in the key resources table.
- Any additional information required to reanalyze the data reported in this paper is available from the lead contact upon request.

### EXPERIMENTAL MODEL AND SUBJECT DETAILS

#### Mice and viruses

For all experiments we used adult (8–16 weeks) male and female mice (two R1Ag5 transgenic mice, B6.Cg-Tg(Camk2a-cre)2Szi/J, The Jackson Laboratory, Jax #027310, and two wild-type mice). Mice were housed on a 12-h light dark cycle in groups of 2–5 mice, and individually after surgery for implantation. Recombinant adeno-associated viruses [rAAV1.Syn.FLEX.GCaMP6f.WPRE.SV4, Addgene #100833] to R1Ag5 mice or [pENN.AAV.CamKII.GCaMP6f.WPRE.SV40, Addgene #100834] to wild-type mice were used for imaging of pyramidal neurons.

All experiments were conducted in accordance with US National Institutes of Health guidelines and with the approval of the Columbia University Institutional Animal Care and Use Committee. No statistical methods were used to predetermine sample sizes. The experiments were not randomized, and the investigators were not blinded to allocation during experiments and outcome assessment.

### METHOD DETAILS

#### Graphene transfer and four-step graphene cleaning protocol

The electrochemical delamination transfer method was adopted to achieve less poly(methyl methacrylate) (PMMA) residue on the surface of the transferred graphene (Thunemann et al., 2018; Lu et al., 2018). 300 nm thick PMMA (Microchem, 495 PMMA A4) was spin coated on chemical vapor deposition (CVD) grown monolayer graphene on copper film (GROLLTEX). In PMMA/graphene/copper layers, copper was connected to the cathode of the DC power supply and the anode was dipped into 0.05M NaOH. With applying 20V DC bias, PMMA/graphene/copper layers were gradually immersed into the NaOH solution with forming hydrogen gas bubbles induced between the graphene and copper layer, which delaminated the PMMA/graphene layers from the copper film. To remove the NaOH residues, the PMMA/graphene layers were transferred to deionized water 3 times. Then, PMMA/graphene layers were transferred on the polyethylene terephthalate (PET) substrate and dried at room temperature. After the PMMA/graphene layers were fully dried, the sample was annealed at 125°C for 5 min to improve graphene/PET adhesion and mitigate the PMMA wrinkles. To remove the PMMA layer, the sample was immersed in acetone for 20 min at room temperature, following by 10 cycles of Isopropyl alcohol (IPA) and deionized water baths (1 min for each cycle).

To reduce the organic residues on the surface of graphene that affect the impedance of graphene electrodes and light-induced artifacts, a four-step cleaning protocol was adopted (Thunemann et al., 2018). After graphene was patterned by using oxygen plasma etching, to remove the protection photoresist layer, the sample was immersed in i) AZ 1-Methyl-2-pyrrolidon (AZNMP) for 10 min and ii) Remover PG for 10 min to remove AZ1512 and PMGI, respectively. To cleanse AZNMP and remover PG, the sample was iii) soaked in acetone for 10min, following by iv) 10 cycles of IPA and deionized water baths. All the processes were conducted at room temperature.

#### Double layer graphene microelectrode array fabrication

To achieve high optical transparency, we used 50 μm-thick PET as a substrate. 20 μm-thick PDMS was spin-coated on a 4-inch silicon wafer to provide mechanical support for PET and attached PET film on top of it. 10 nm Chromium and 100 nm gold were sputtered onto the PET substrate film using Denton Discovery 18 Sputter System. Metal wires and contact pad for zero insertion force (ZIF) connector were patterned with photolithography and wet-etching. The first graphene layer was transferred by using the bubbling transfer method as described above. After cleaning the first graphene layer, the second layer graphene was transferred and cleansed with the same procedure as the first layer graphene. To minimize the photoresist residue and protect the mechanical damage during patterning the double-layer graphene, PMGI/AZ1512 bilayer photoresist was applied on the double-layer graphene: i) 100 nm PMGI SF3 was spin-coated at 3000 rpm for 45 s and baked at 125°C for 5 min and ii) 1.2 μm AZ1512 was spin-coated at 4000 rpm for 45 s and baked at 95°C for 1 min. This bilayer photoresist was patterned with photolithography and double-layer graphene was etched with oxygen plasma etching (Plasma Etch PE100), following by a four-step graphene cleaning protocol as described above. To define the neural signal recording area, a 3-μm thick SU-8 encapsulation layer was spin-coated at 1500 rpm for 45 s and baked at 95°C for 2 min. This encapsulation was patterned with photolithography and developed with SU-8 developer for 1min. To remove the SU-8 residue on the opening or recording graphene area, the sample was rinsed with 10 cycles of IPA and deionized water baths.

#### Surgical procedures

Stereotaxic rAAV injections were performed with a Nanoject syringe, as previously described (Kaufman et al., 2020). Mice were anesthetized with isoflurane and treated with 0.1 mg/kg buprenorphine or meloxicam for analgesia and 180nL (60nL per spot) of diluted virus (1:2 in sterile cortex buffer) was injected into left dorsal hippocampal CA1 (from bregma AP −2.2, ML −1.75, and DV −1.0, −1.1, and −1.2 mm). E-Cannula was constructed by a graphene microelectrode, a 3-mm diameter cover glass (64-0720, Warner), and a stainless-steel cannula (bottom face: 3-mm diameter circle, 1.5-mm height, 45-angle of inclination at one side). The cover glass was first attached to the cannula with optical adhesive (Norland optical adhesive 81). Then we applied a thin layer of optical adhesive to the surface of the cover glass and attached the graphene microelectrode array. The shank of the graphene microelectrode array was also attached to the wall of the cannula with optical adhesive. Finally, we used ultraviolet light to cure the adhesive until all the components were held in place. After 3–4 days for recovery, mice were implanted with E-Cannula over the left dorsal hippocampus as well as a steelhead-bar for head-fixation. The scalp was removed, a 3.0 mm diameter craniotomy centered over the injection location was performed using a fin-tipped dental drill, and then the lateral side (~1.0 mm at 2.35 rad) was sculpted to precisely match the size of the E-Cannula base. The dura was removed, and the underlying cortex aspirated until fibers within the alveus were visible, while the chilled cortex buffer was constantly irrigated. Stopping any residual bleeding with gel foam, we gently wedged E-Cannula into the craniotomy and lowered until it tightly touched the alveus fibers. After the cannula was glued to the skull, we bent electrode wire, attached it to the cannula inclination, and covered all components with dental cement. A stainless-steel jewelers screw served as a ground/reference were inserted into the cerebellum.

#### *In vivo* two-photon imaging and electrophysiological recording

After recovery from surgery, mice were handled for several days, and habituated to head-fixation and experimental setup. Mice were subsequently trained to run on the treadmill. Multimodal recording experiments started at 10 days following implantation surgery.

All imaging was conducted using a two-photon 8×kHz resonant scanner (Bruker) and 16× NIR water immersion objectives (Nikon, 0.8 NA, 3.0-mm working distance, respectively). We acquired 840 × 840 μm^2^ images (512 x 512 pixels) at 30 Hz using a 920-nm laser (50–100 mW, Chameleon Ultra II, Coherent) from CA1 stratum pyramidale. Green (GCaMP) fluorescence was detected with a GaAsP PMT (Hamamatsu Model 7422P-40). The preprocessing steps for acquired fluorescence signal using the SIMA software package were described in our previous work (Ahmed et al., 2020). After SIMA-based motion correction, we detected neural regions of interest (ROIs), extracted associated fluorescence signals, and calculated neuropil-decontaminated ΔF/F_0_ using the Suite2p software package with built-in neuropil subtraction (Pachitariu et al., 2016). Identified ROIs were curated post hoc using the Suite2p graphical interface to exclude non-somatic components.

The microelectrode array of the E-cannula was connected to a customized connector board that routed the electrical signals to the Intan RHD2132 amplifier. Electrophysiological recordings were done with a multichannel recording system (Intan Technologies) synchronized with the AOD imaging system. The data was recorded at 20 kHz. In total, four mice were recorded.

#### SWR detection and spike sorting

The detection of SWRs was performed by the following procedures (Liu et al., 2021). The raw recording signals were first band-pass filtered between 120 and 250 Hz (4th order Butterworth filter) in both forward and reverse directions to prevent phase distortion. Then we applied Hilbert transform to extract the envelope of the ripple-band signals. To detect the potential SWR events in a single channel, we set a threshold to 4.5 standard deviations above the mean. When the ripple envelope crossed the threshold, a candidate SWR event was labeled, and its start and end were further defined as the time when the ripple envelope returned back to the mean level. Similar to the previous study, we merged the two candidate SWR events whose inter-event interval was less than 10 ms and discarded the candidate SWR events with duration less than 20 ms. After we identified the SWR events detected in each individual recording channel, we further defined the array-level SWR events. The start time was chosen as the earliest start time in all the channels detecting SWR, whereas the end time was chosen as the latest end time in all the channels detecting SWR. Finally, for all the automatically detected array-level SWR events, we further performed manual curation to discard the artifacts.

The spike sorting was performed with Kilosort 2 (Pachitariu et al., 2016). Before the spike sorting, all of the recordings from the same day were put together to find the same putative spikes across these recordings. The Kilosort algorithm then determined the best templates and potential spike clusters, as well as their spike timing and amplitudes. Then we performed manual curation with Phy (Rossant et al., 2016) software to refine the clustering results by checking the spike waveforms and the auto- and cross-correlograms. We also performed merge and split as needed to obtain higher-quality clusters. After rejecting the clusters corresponding to noise, we labeled the rest of the good clusters as multi-unit (MUA) since their spike waveforms resembled typical action potential waveforms, but their auto-correlograms exhibited certain violations of the refractory period.

#### Detection of synchronous calcium events and co-occurrence with SWR

To detect the synchronous calcium events (SCEs), we followed similar approach in previous study (Malvache et al., 2016) with slight modifications. After computing the deconvolved spikes of all the imaged cells, we used thresholds of 3 standard deviation to define the firing intervals for each cell. Then for each time frame, we count the number of firing cells in the adjacent 5 time frames (~160 ms). To obtain the chance level firing cell number, we circularly shuffled the firing intervals 20 times for each cell and pooled the resulting firing cell number at all time frames. The 95 percentiles of the shuffled was chosen as the threshold. Then we applied this threshold to the number of firing cells at each frame in the original data to identify the time of SCEs. When computing the co-occurrence of the SCEs and the SWRs, we used a 200 ms time window. When a SWR and a SCE occur within 200 ms, they were labeled as co-occurring. To determine the significance of the SWR-to-SCE and SCE-to-SWR co-occurrence ratios, the timing of SWR were randomly permutated 1000 times and the p values were computed as the percentage of resulting chance-level co-occurrences that were greater than the original co-occurrence.

#### Clustering of SWR events

Before the clustering, we need to compute the spatiotemporal features of SWR events. To do that, we first computed the envelopes of the ripple band (120–250 Hz) signals from all the channels and computed the cross-correlation between each envelope and the mean envelope across all the 16 channels to determine the channel-wise time delay. For any channel, if the maximum cross-correlation is less than 0.5, we did not attempt to evaluate its time delay. These situations mainly happened when the ripple signals in that channel exhibited complex envelopes so that the accurate determination of its timing is difficult. If the number of channels with evaluated time delay is less than 13, we discarded that event. Otherwise, we performed interpolations to fill in the time delays for the missing channels. To capture the spatial activation profile of the SWR events, we computed the magnitude of ripple band power at each channel and normalized it with the maximal value among all the channels. Finally, we computed the difference between the envelope in each channel and a standard Gaussian curve using dynamic time warping and discarded the SWR events with complex envelope shapes (distance >3.5 SD of ripple envelopes) and only focused on the SWR events with single dominant peak in their envelopes. These time delays and the normalized power magnitudes were then scaled to have the same standard deviations and used as features to capture the spatiotemporal characteristics of the SWR events recorded in the 2D electrode grid.

With the above features, we performed K-means clustering to assign each SWR event to different ripple cluster types. To determine the proper number of clusters, we computed the within-cluster sum of squared distances under the different number of clusters and chose the “elbow” point. We repeated the K-means clustering 10 times and chose the best clustering results. The template for each ripple cluster was obtained by averaging over all the SWR events assigned to the same cluster. Finally, we further grouped the identified ripple clusters into four bigger groups (stationary-local, stationary-global, traveling-local and traveling-global) depending on whether the maximum channel-wise time delays and the of the ripple templates. The ripple clusters with maximum channel-wise time delay greater than 5 ms were considered as traveling. Otherwise, they were defined as stationary. For the template of each ripple cluster, we defined each channel as active if its normalized power magnitude was greater than 0.6. The ripple clusters with more than 10 active channels were considered as global. Otherwise, they were defined as local.

#### The algorithm for decoding the SWR events with different spatiotemporal characteristics

To decode the ripple cluster types using the cellular activity of hippocampal neurons, we used the support vector machine (SVM). The mean ΔF/F activity of each neuron during the [−150 ms, +150ms] around SWR onset were used as input features for the SVM algorithm. Since different ripple clusters have the different number of SWR events, the resulting dataset is unbalanced. To overcome this difficulty, we created a new balanced dataset by subsampling the SWR events of different ripple clusters to match the number of SWR events in the smallest ripple cluster. Then the SVM model was trained on this new balanced dataset. To identify the most discriminative neurons and prevent overfitting, we performed recursive feature elimination (Diamantaki et al., 2018) with a step size of 50 neurons at a time. To measure the decoding performance, we evaluated the accuracy, precision, and recall using 10-fold cross-validation and compared these metrics against the results given by a random classifier.

#### Detection of cell assembly and the firing ratio under different ripple clusters

To detect the cell assembly, we first computed the ΔF/F of cells between [−150 ms, +150ms] around each ripple onset. Then we applied a threshold of 30% on the mean ΔF/F of each cell to label it as active or silent during each SWR event. By computing the Jaccard similarity between each pair of neurons, we obtained the similarity matrix. To determine the chance level similarity, we construct a surrogate dataset by shuffling the identity of the active SWR events for each cell and recomputed the similarity matrix. By repeating this process for 200 times, we obtained a null distribution of the chance level similarity between each neuron pair. We then set any pairwise similarity below that threshold to zero. Using thresholded similarity matrix for both the original dataset and the surrogate dataset, we performed clustering using a community detection algorithm to identify the putative cell assemblies. In the surrogate data, we found that many small-sized cell assemblies could emerge randomly. Therefore, we defined a threshold on the size of cell assembly as the 99th percentile of the cluster size obtained from the surrogate dataset. We then used this threshold to discard any putative cell assemblies in the original dataset whose sizes are smaller. The clusters that survive all these criteria were treated as identified cell assemblies.

For the control analysis to evaluate the effect of assembly relationship on decoding of SWRs we compared the decoding performance under two conditions. As illustrated in Figure S7G (left), for the first condition (Holding), we randomly sampled an increasing number of cells from all assemblies (step = 5%) and used their activities as input features for the SVM model. For the second condition (In-order), we sequentially selected an equal number of cells (as in condition one) and performed the decoding. We repeated the above analysis 200 times and statistically compared the decoding accuracies of two conditions.

### QUANTIFICATION AND STATISTICAL ANALYSIS

All statistical analyses were performed in MATLAB. Statistical tests were two-tailed and significance was defined by p value pre-set to 0.05. The error bars and shaded regions around line-plots represent ±standard error of the mean (s.e.m.) unless otherwise noted. All the statistical tests are described in the figure legends and each test was selected based on data distributions using histograms. Detailed statistical procedures are described in each sub-section of STAR Methods. Multiple comparisons were corrected for by Benjamini-Hochberg corrections.

## Supplementary Material

1

## Figures and Tables

**Table:** KEY RESOURCES TABLE

REAGENT or RESOURCE	SOURCE	IDENTIFIER
Bacterial and virus strains		
pAAV1.Syn.FLEX.GCaMP6f.WPRE.SV4	Addgene	RRID:Addgene100833
pENN.AAV.CamKII.GCaMP6f.WPRE.SV40	Addgene	RRID: Addgene_100834
Experimental models: Organisms/strains		
R1Ag5 mice: B6.Cg-Tg(Camk2a-cre)2Szi/J	The Jackson Laboratory	Jax #027310
Software and algorithms		
Suite2p	Pachitariu et al., 2017	https://github.com/cortex-lab/Suite2P
SIMA	Kaifosh et al., 2014	https://github.com/losonczylab/sima
Python 3.7	Python Software Foundation	https://www.python.org
MATLAB	MathWorks	https://www.mathworks.com/
Custom code	This paper	https://github.com/mehrdadramezani/E-Cannula (https://zenodo.org/record/7055600)

## Highlights

- Two-photon imaging and electrophysiological recording from the same hemisphere of CA1
- Multiunit spiking activity phase locked to local ripples
- Local ripple generation and propagation along the septotemporal and transverse axes
- Distinct spatiotemporal ripple patterns recruiting orthogonal cell assemblies
