# Supplementary material for: E-Cannula reveals anatomical diversity in sharp-wave ripples as a driver for the recruitment of distinct hippocampal assemblies
Source: Cell Rep. Author manuscript; Available in PMC 2022 Nov 7. (PMC9640218; doi:10.1016/j.celrep.2022.111453)
Supplement: 1 [file NIHMS1840778-supplement-1.pdf]

Cell Reports, Volume *41*

Supplemental information

E-Cannula reveals anatomical diversity in sharp-wave ripples as a driver for the recruitment of distinct hippocampal assemblies

Xin Liu, Satoshi Terada, Mehrdad Ramezani, Jeong-Hoon Kim, Yichen Lu, Andres Grosmark, Attila Losonczy, and Duygu Kuzum

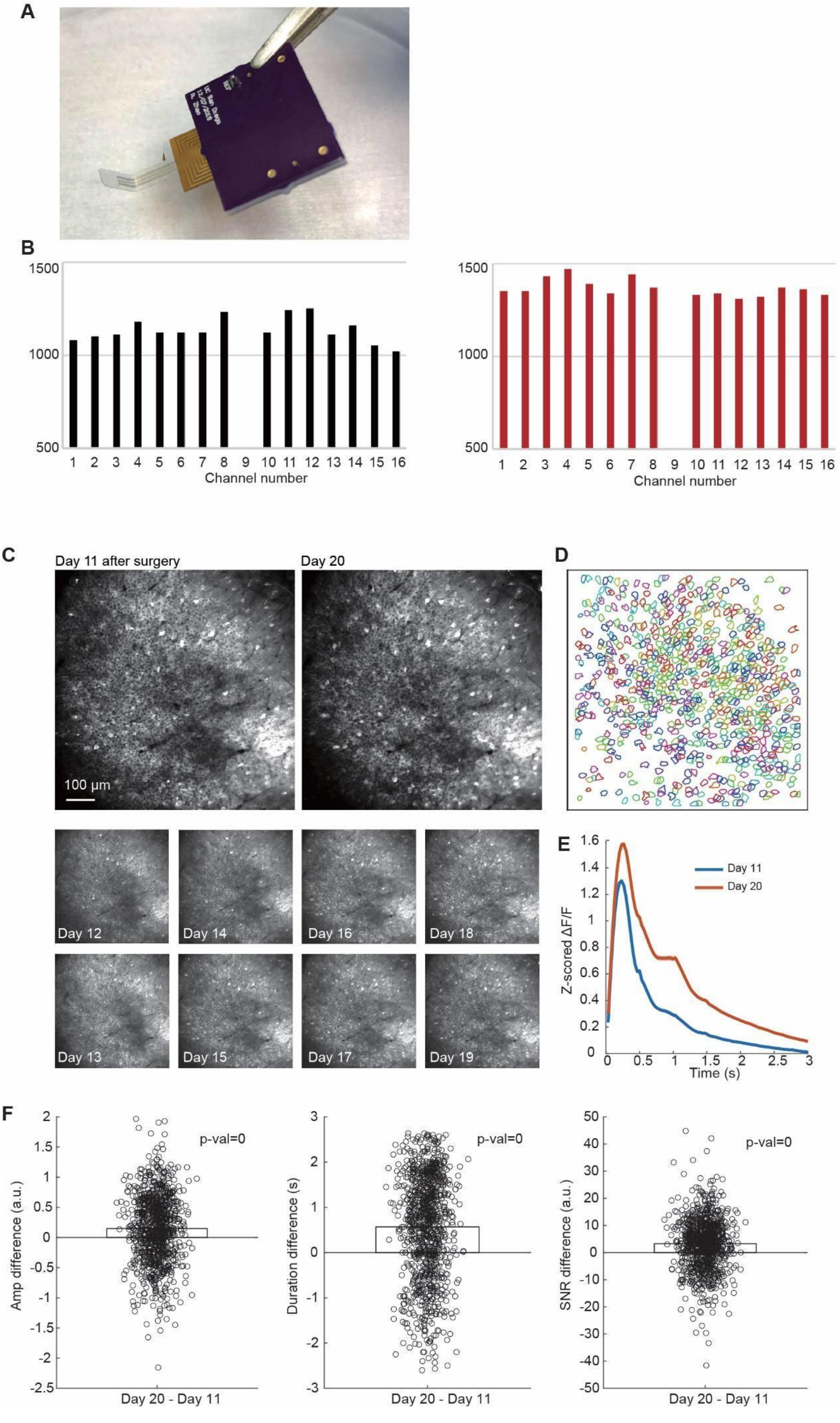
A
B
1500
1000
500
1 2 3 4 5 6 7 8 9 10 11 12 13 14 15 16
Channel number
1500
1000
500
1 2 3 4 5 6 7 8 9 10 11 12 13 14 15 16
Channel number
C
Day 11 after surgery
Day 20
100 µm
Day 12
Day 14
Day 16
Day 18
Day 13
Day 15
Day 17
Day 19
D
E
Day 11
Day 20
Z-scored ΔF/F
1.6
1.4
1.2
1
0.8
0.6
0.4
0.2
0
0 0.5 1 1.5 2 2.5 3
Time (s)
F
Amp difference (a.u.)
2
1.5
1
0.5
0
-0.5
-1
-1.5
-2
-2.5
p-val=0
Day 20 - Day 11
Duration difference (s)
3
2
1
0
-1
-2
-3
p-val=0
Day 20 - Day 11
SNR difference (a.u.)
50
40
30
20
10
0
-10
-20
-30
-40
-50
p-val=0
Day 20 - Day 11

Figure S1. Comparison of imaging data obtained with E-cannula on Day 11 and Day 20. (related to Figure 1 and 2)

(A) Picture of the electrode array after 90 degrees bending.

(B) The electrode impedance before bending (top) and after bending (bottom). The electrode array still works with the impedance slightly increased after the excessive bending.

(C) Example imaging field of view and region of interests obtained at Day 11 and Day 20 after viral injection (WT mouse injected with CamKII promoter virus).

(D) Suite2p-detected ROIs from (A).

(E) The mean $\Delta F/F$ response across cells on Day 11 (blue) and Day 20 (red).

(F) The comparison of the amplitude, duration, and SNR of GCaMP-calcium events in Day 11 and 20.

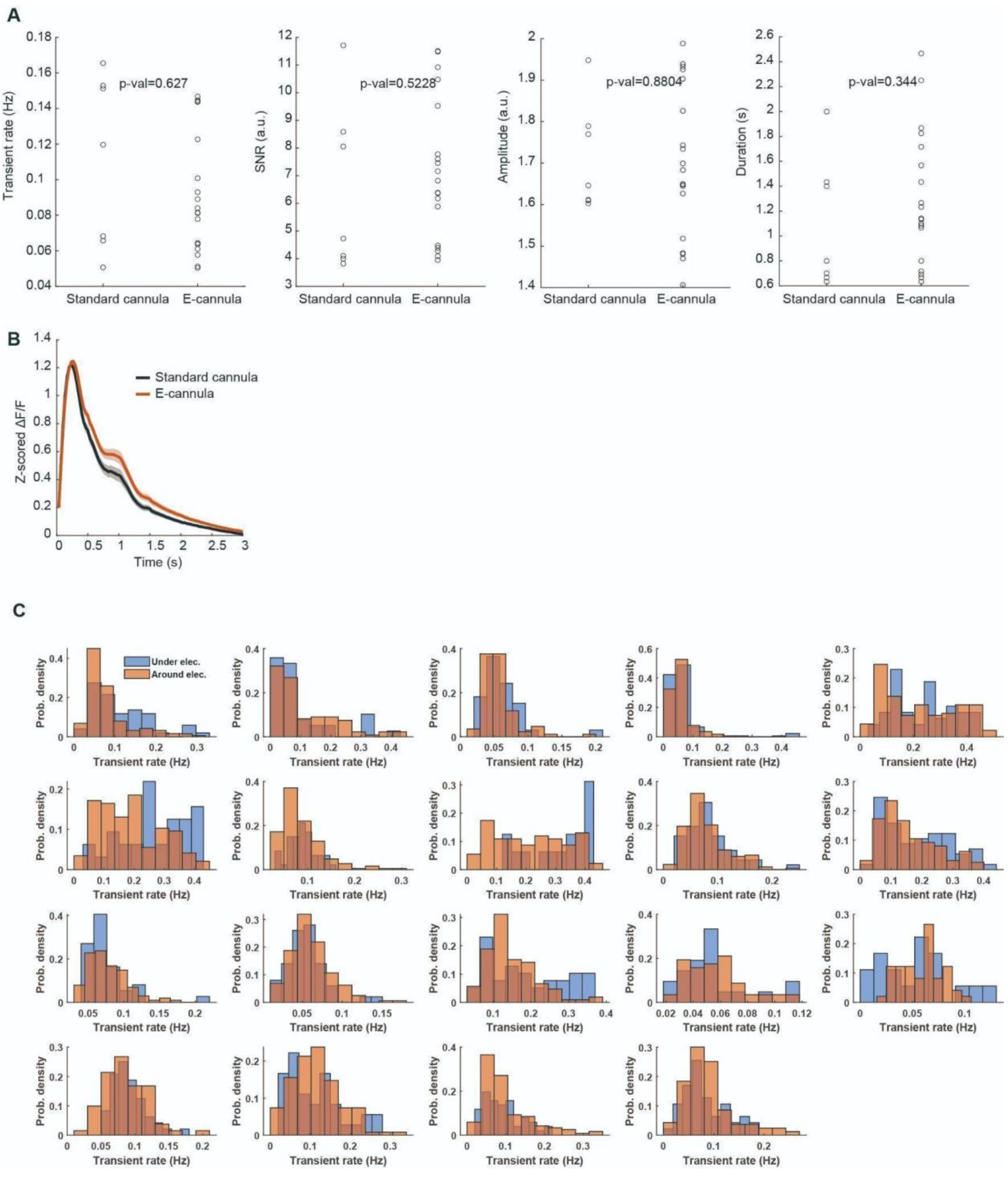

Figure S2. Comparison of imaging data obtained with standard cannula and the E-cannula. (related to Figure 2)

(A) GCaMP-calcium transient rate, SNR, amplitude, and duration of the ΔF/F response, showing similar data quality between the standard cannula and the E-cannula.

(B) The mean ΔF/F response obtained with standard cannula and the E-cannula. The responses are similar (cluster-based permutation test).

(C) Transient rate distribution of cells under the electrode (blue) and around the electrode (orange) for 19 recording sessions in 3 mice.

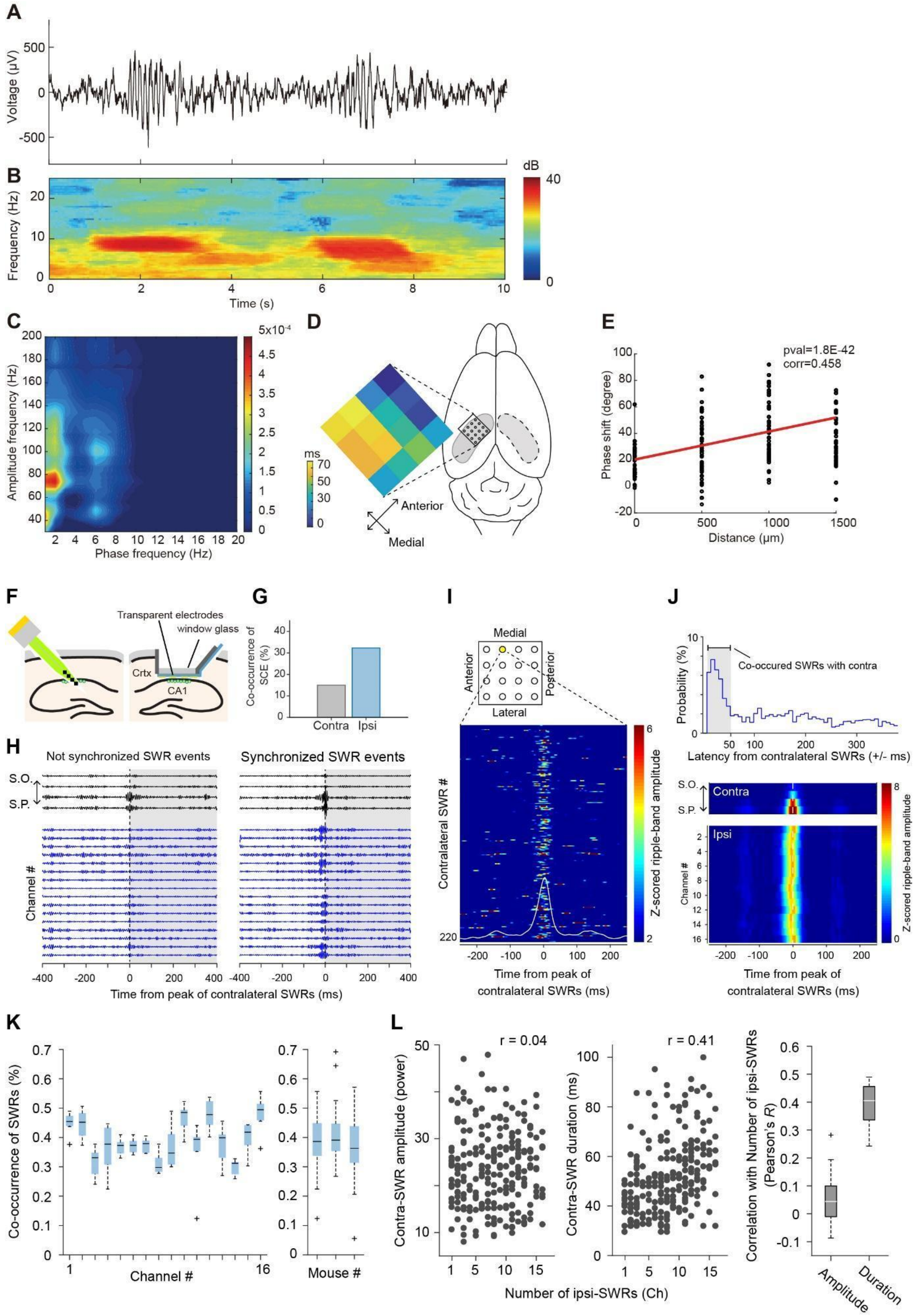

A
Voltage (μV)
B
Frequency (Hz)
dB
Time (s)
C
Amplitude frequency (Hz)
Phase frequency (Hz)
D
ms
Anterior
Medial
E
pval=1.8E-42
corr=0.458
Phase shift (degree)
Distance (μm)
F
Transparent electrodes
window glass
Crtx
CA1
G
Co-occurrence of SCE (%)
Contra
Ipsi
H
Not synchronized SWR events
Synchronized SWR events
S.O.
S.P.
Channel #
Time from peak of contralateral SWRs (ms)
I
Medial
Anterior
Posterior
Lateral
Contralateral SWR #
Z-scored ripple-band amplitude
Time from peak of contralateral SWRs (ms)
J
Probability (%)
Co-occured SWRs with contra
Latency from contralateral SWRs (+/- ms)
Contra
Ipsi
Channel #
Z-scored ripple-band amplitude
Time from peak of contralateral SWRs (ms)
K
Co-occurrence of SWRs (%)
Channel #
Mouse #
L
r = 0.04
r = 0.41
Contra-SWR amplitude (power)
Contra-SWR duration (ms)
Number of ipsi-SWRs (Ch)
Correlation with Number of ipsi-SWRs (Pearson's R)
Amplitude
Duration

Figure S3. Co-occurrence of SWRs recorded bilaterally with one linear silicon probe and E-Cannula in each hippocampus. (related to Figure 3)

(A) Example LFP recordings from one channel showing two theta events (WT mouse injected with CamKII promoter virus).

(B) Spectrogram for recordings in a, showing power increase in theta band.

(C) Phase-amplitude coupling plot, showing significant theta-gamma and delta-gamma coupling in the electrical recordings.

(D) phase shift of theta band activity across array for one theta event (left) and the implantation map, showing obvious traveling of theta waves across the septotemporal axis. Corr = 0.458, P < 0.001. Traveling speed = 0.148m/s.

(E) The phase shift of theta band activity as a function of septotemporal distance, showing a clear trend.

(F) The schematics of bilateral LFP recording with two-photon imaging. The same procedure of E-cannula implantation shown as Figure 2A, and 4 channel linear silicon probe was implanted into contralateral CA1. We assessed the layers where each probe channel was located according to the polarity of the sharp-wave and the existence of ripples and used the channel showing most prominent ripples to detect the peak time of SWRs for the following analysis.

(G) Co-occurrence of SCEs with ipsilateral (ipsi-) and contralateral (contra-) SWRs

(H) Two representative examples to show “not-synchronous” and "synchronous” SWR events. The lines indicate LFPs recorded from contralateral silicon probe (black) and E-cannula (blue). S.O., stratum oriens, S.P., stratum pyramidale.

(I) Z-scored ripple-band envelope amplitudes around contra-SWRs in a session (white: average amplitude). The top cartoon indicates the channel location on the array.

(J) Top, distribution of ipsi-SWR occurrence latencies (n= 269.68 ± 23.6 contra-SWRs per session from 3 mice, mean ± s.e.m). We defined “Co-occurred SWRs” as the ipsi-SWRs occurring within 50ms from the peak of contra-SWRs (SWRs in the gray area). Bottom, an example of average z scored ripple-band envelope amplitudes around contra-SWRs (top: recorded from contralateral silicon probes with 4 ch, bottom: from E-cannula with 16 ch).

(K) Left, the proportions of co-occurred SWRs from each channel in a mouse (n = 4 sessions from a mouse). Right, mean proportions across the 16 channels in all 3 mice (n = 4 sessions per mouse, 38.64% ± 0.01, median ± s.e.m). Black center line in the box plot indicates the median, bottom and top edges of the box denote 25th and 75th percentiles, and the cross symbol means outliers.

(L) An example of scatter plots for all pairs of the number of co-occurred SWRs and the amplitudes (left), or the durations (middle) of contra-SWRs in a session. Right, Pearson’s linear correlation coefficients for all pairs in all 12 sessions from 3 mice.

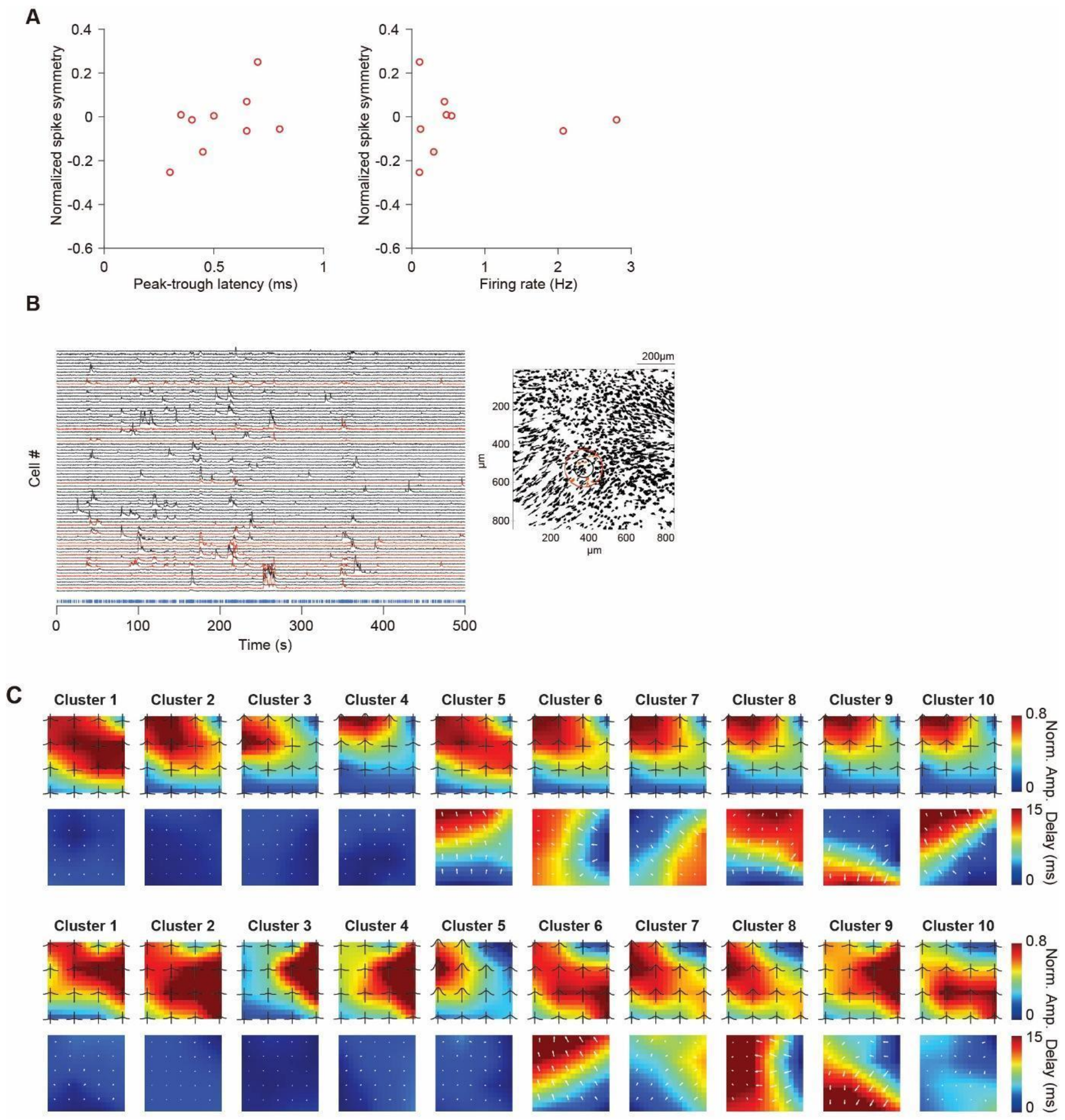

Figure S4. Waveform characteristics of MUA clusters and the simultaneously recorded fluorescence activity of cells. (related to Figure 4 and 6)

(A) Each dot represents one detected MUA cluster. n=4 mice.

(B) The matching between cellular fluorescence activity and the MUA activity. (left) The ΔF/F activity of cells within 100 μm to the electrode center and the spike raster plot for the MUA cluster from the same electrode. The cells showing significant matching are labeled as red. (right) The location of the cells within the FOV. The cells showing significant matching are labeled as red.

(C) Identified ripple clusters showing different spatial activation patterns (top) and temporal delays (bottom) detected from the rest of two mice.

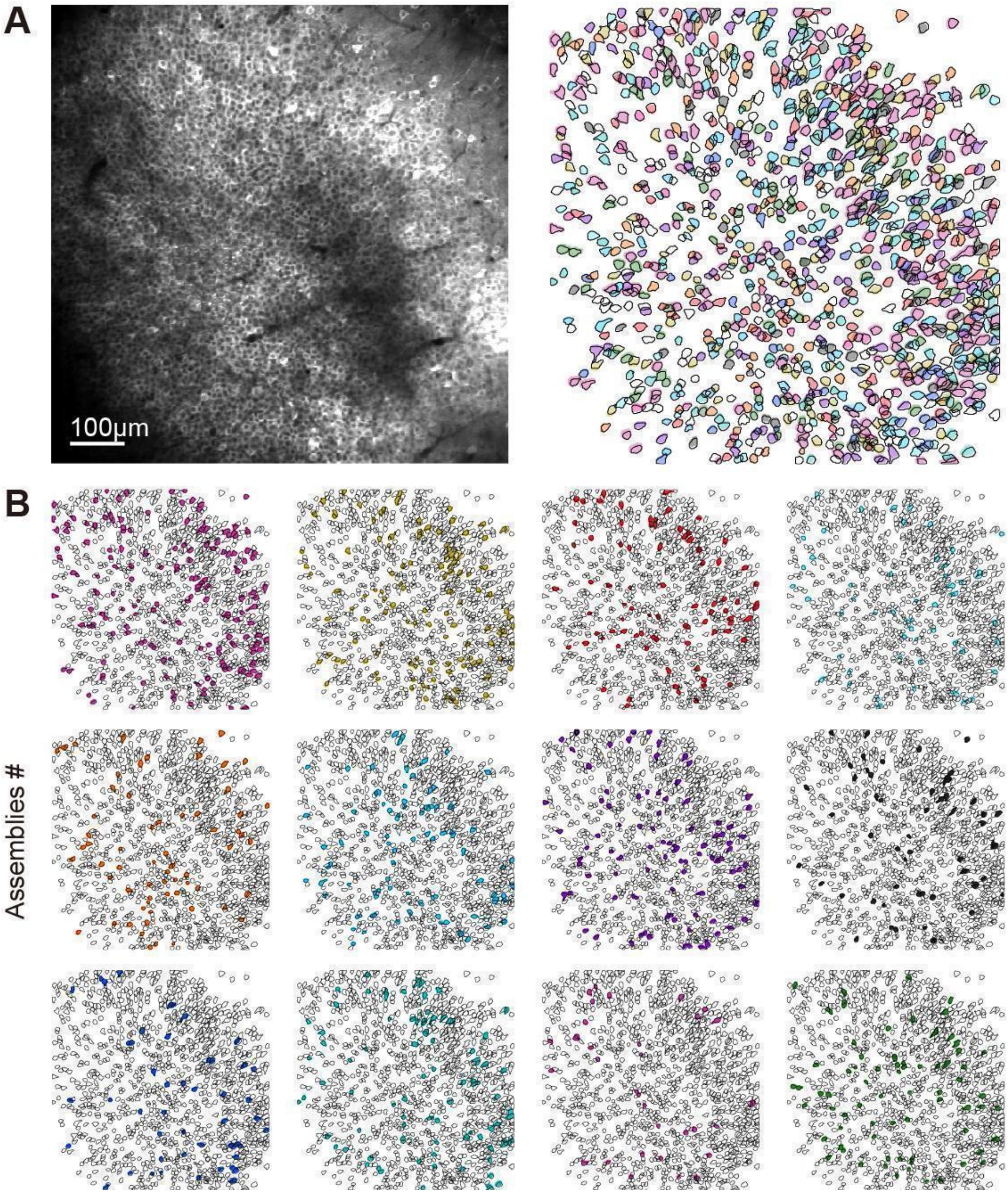

Figure S5. Additional data on topological distribution of cells within discrete cell assemblies. (related to Figure 7)

(A) Representative FOV and ROI map of the imaged cells. Scale bar = 100 μm (WT mouse injected with CamKII promoter virus). Filled colored ROIs illustrate the cells participating in one of the assemblies, and each color indicates different assembly participations.

(B) all topological maps of the cells participating in each assembly. The left top and right bottom maps are shown in Figure 5B.

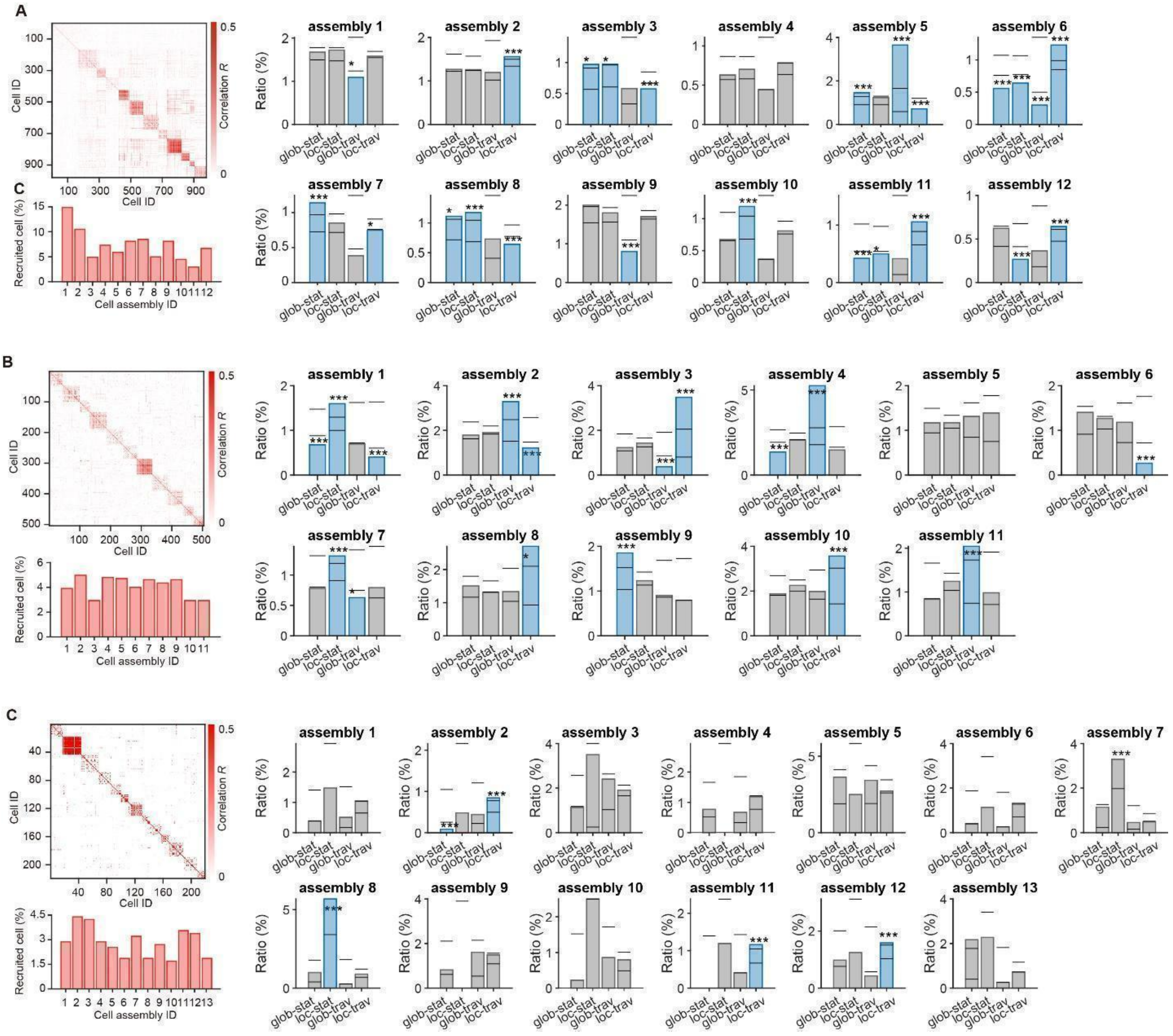

Figure S6. Activity of hippocampal cell assemblies during different ripple clusters for different mice. (related to Figure 7)

(A) Adjacency matrix of the identified cell assemblies, the ratio of cells assigned to each assembly, and the firing ratio of cell assemblies under different ripple clusters. The ratio of each cell assembly under each ripple cluster was tested by shuffling test ($P < 0.05$ with FDR correction, $^*P < 0.05$, $^{**}P < 0.01$, $^{***}P < 0.001$). The bars are the 2.5 percentile and 97.5 percentile values obtained from shuffled data (CamKII-cre mouse injected with FLEX virus).

(B) Same as in (A) with data from the second mouse (WT mouse injected with CamKII promoter virus).

(C) Same as in (A) with data from the third mouse (WT mouse injected with CamKII promoter virus).

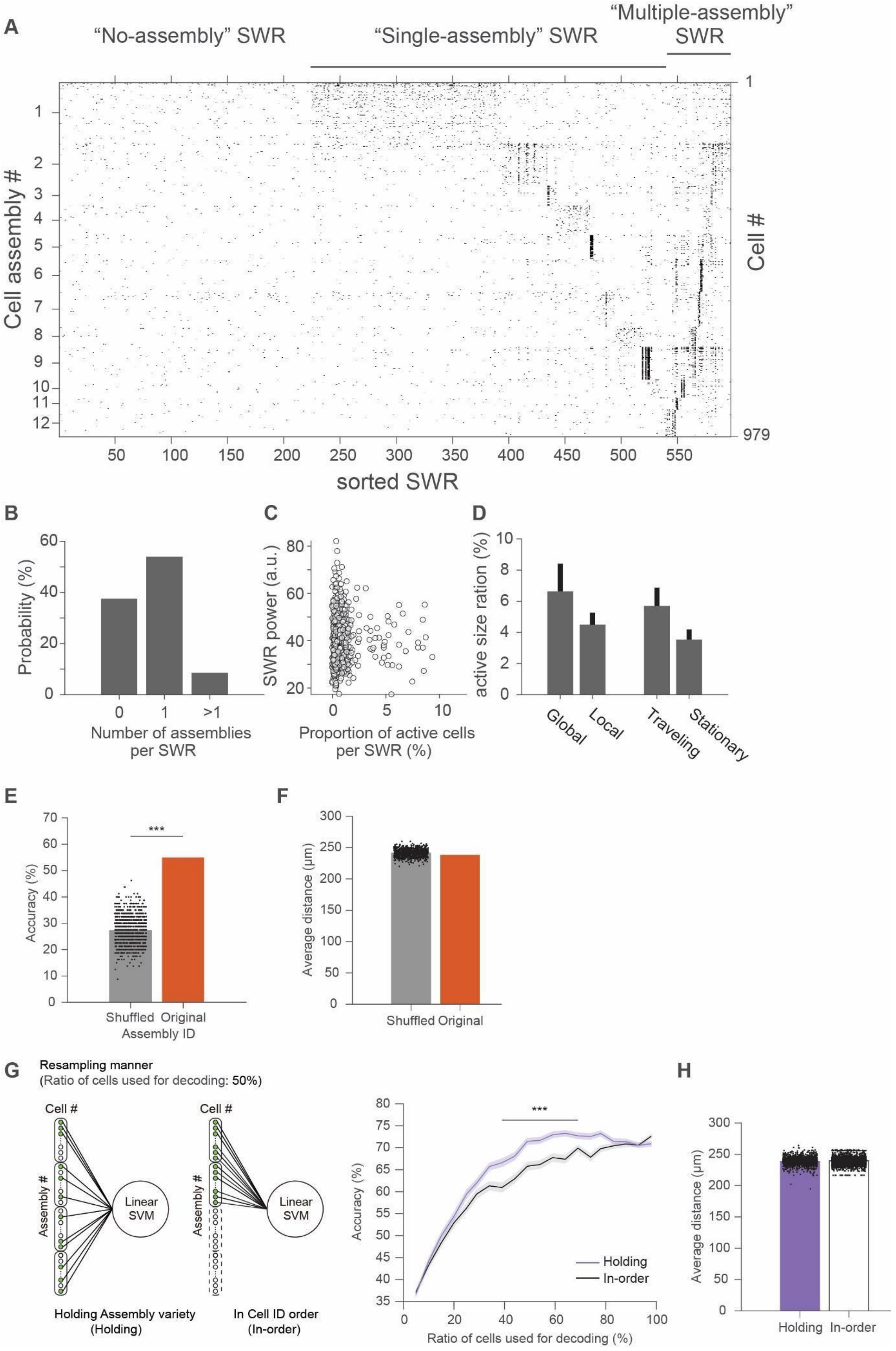

Figure S7. Relationship between SWRs and recruited cell assemblies. (related to Figure

6 and 7)

(A) raster plot of cells sorted by SWRs and their selection of cell assemblies. Three clusters of SWRs are identified based on their cell assembly selection.
(B) number of cell assemblies recruited during each SWR.
(C) scatter plot between participation of cells and the SWR power (n = 597 SWR events). No significant correlation found (r = 0.07, P = 0.1, Spearman).
(D) ratio of active cells from assemblies preferring different types of SWRs (global/local and traveling/stationary). The error bars indicate the s.e.m.
(E) control analysis to investigate the effect of population dispersion on decoding SWRs. decoding accuracy using original assembly labels is significantly higher than the shuffled labels. (Two-tailed t-test, ***P < 0.001).
(F) average pairwise distance of cell populations used for the control analysis in (A).
(G) control analysis to show the effect of assembly relationship on decoding of SWRs. Left, schematics of resampling methods for two conditions (Holding and In-order). *Right*, decoding accuracy of both conditions (200 trials) for increasing number of sampled cells (total number of cells is 979, same cells from A). The bold line is the mean accuracy, and the shaded region indicates the s.e.m. Wilcoxon rank-sum tests (****P* < 0.001).
(H) average pairwise distance of cell populations used for the control analysis in (C).
